# An Optogenetic‐Controlled Cell Reprogramming System for Driving Cell Fate and Light‐Responsive Chimeric Mice

**DOI:** 10.1002/advs.202202858

**Published:** 2022-12-11

**Authors:** Meiyan Wang, Yuanxiao Liu, Ziwei Wang, Longliang Qiao, Xiaoding Ma, Lingfeng Hu, Deqiang Kong, Yuan Wang, Haifeng Ye

**Affiliations:** ^1^ Shanghai Frontiers Science Center of Genome Editing and Cell Therapy Biomedical Synthetic Biology Research Center Shanghai Key Laboratory of Regulatory Biology Institute of Biomedical Sciences and School of Life Sciences East China Normal University Dongchuan Road 500 Shanghai 200241 China; ^2^ Department of Animal Sciences, College of Agriculture and Natural Resources Michigan State University East Lansing MI 48824 USA

**Keywords:** cell reprogramming, chimeric mice, endogenous transcription factors, optogenetics, regenerative medicines

## Abstract

Pluripotent stem cells (PSCs) hold great promise for cell‐based therapies, disease modeling, and drug discovery. Classic somatic cell reprogramming to generate induced pluripotent stem cells (iPSCs) is often achieved based on overexpression of transcription factors (TFs). However, this process is limited by side effect of overexpressed TFs and unpredicted targeting of TFs. Pinpoint control over endogenous TFs expression can provide the ability to reprogram cell fate and tissue function. Here, a light‐inducible cell reprogramming (LIRE) system is developed based on a photoreceptor protein cryptochrome system and clustered regularly interspaced short palindromic repeats/nuclease‐deficient CRISPR‐associated protein 9 for induced PSCs reprogramming. This system enables remote, non‐invasive optogenetical regulation of endogenous *Sox2* and *Oct4* loci to reprogram mouse embryonic fibroblasts into iPSCs (iPSC_LIRE_) under light‐emitting diode‐based illumination. iPSC_LIRE_ cells can be efficiently differentiated into different cells by upregulating a corresponding TF. iPSC_LIRE_ cells are used for blastocyst injection and optogenetic chimeric mice are successfully generated, which enables non‐invasive control of user‐defined endogenous genes in vivo, providing a valuable tool for facile and traceless controlled gene expression studies and genetic screens in mice. This LIRE system offers a remote, traceless, and non‐invasive approach for cellular reprogramming and modeling of complex human diseases in basic biological research and regenerative medicine applications.

## Graphical abstract

A light‐inducible cell reprogramming (LIRE) system is developed based on the photoreceptor protein phytochrome and clustered regularly interspaced short palindromic repeats/nuclease‐deficient CRISPR‐associated protein 9, which enables non‐invasive, precise regulation of endogenous *Sox2* and *Oct4* to initiate pluripotent reprogramming under light illumination. Moreover, iPSC_LIRE_ cells can be differentiated into different cell types by illumination and can generate the optogenetic chimeric mice which will uncover unknown gene functions in vivo.

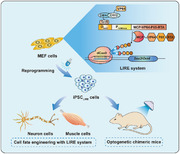

## 1 Introduction

Pluripotent stem cells (PSCs) hold great promise for autologous cell‐based therapies, disease modeling, and drug discovery.^[^ ^1^, ^2^, ^3^, ^4^, ^5^, ^6^, ^7^ ^]^ Conventionally, somatic cells can be reprogrammed into induced pluripotent stem cells (iPSCs) by forced overexpression of the ectopic transcription factors (OSKM TFs): Oct4 (POU factor), Sox2 (a member of the Sox (SRY‐related HMG box) gene family), Klf4 (KrÜppel‐like factor 4), and c‐Myc (a member of the basic helix‐loop‐helix leucinezipper (bHLHZip) protein family).^[^ ^8^, ^9^, ^10^, ^11^, ^12^, ^13^ ^]^ Despite great progress, this strategy faces various challenges. For example, overexpressed TFs have side effect due to disturbed stoichiometry^[^ ^14^ ^]^ and lack of precise control over the dose and timing of TFs leads to off‐target gene activation, which caused emergence of alternative cell types and aberrant iPSCs and then hamper further application of iPSCs.^[^ ^15^ ^]^ Moreover, if the endogenous pluripotent gene transcriptional network is not fully activated by the exogenous TFs, incomplete reprogramming can occur, which could result in undesired downstream differentiation.^[^ ^16^, ^17^, ^18^ ^]^

Recently, a revolutionary gene targeting technology, the clustered regularly interspaced short palindromic repeats (CRISPR) and nuclease‐deficient CRISPR‐associated protein 9 (dCas9) technology, has been repurposed as a powerful tool for programmable genome manipulation in eukaryotic cells,^[^ ^19^, ^20^, ^21^, ^22^, ^23^, ^24^, ^25^ ^]^ which provides an effective method for activating the endogenous transcriptional networks involved in reprogramming, as an alternative to delivering exogenous reprogramming factors.^[^ ^26^, ^27^ ^]^ However, current programmable genome editing technologies typically rely on constitutive expression of endogenous genes, which offers no capacity for temporal control of gene expression. Therefore, these modalities remain less suitable for therapeutic cell and organismal engineering. Of note, precisely regulating the timing and dosage of TFs affects cellular reprogramming, because sustained high TF levels appear to hinder the proper establishment of specific chromatin marks during the later stages of iPSC induction.^[^ ^28^, ^29^ ^]^ Therefore, an intelligent cellular reprogramming method with traceless and non‐invasive control over TFs expression would be welcomed.

At present, light has become a widely used exogenous trigger for endogenous gene expression, owing to its superiorities of non‐invasiveness and high spatiotemporal resolution and its avoidance of some drawbacks with chemical inducers.^[^ ^30^, ^31^, ^32^, ^33^, ^34^, ^35^, ^36^, ^37^, ^38^, ^39^, ^40^, ^41^ ^]^ Previously, several light‐inducible CRISPR‐dCas9 transcription systems—based on CRY2/CIB1,^[^ ^42^ ^]^ positive Magnet (pMag)/negative Magnet (nMag),^[^ ^43^ ^]^ and BphS^[^ ^37^ ^]^—have been developed to induce target gene activation for cell differentiation, but to the best of our knowledge, no optogenetically controlled CRISPR/dCas9 system for multiple gene activation for complete pluripotent reprogramming has been developed. Moreover, there are some drawbacks with existing light‐inducible CRISPR‐dCas9 transcription systems, including high background activity of pMag/nMag‐based photoactivatable transcription system (split‐CPTS 2.0)^[^ ^43^ ^]^ and the relatively complicated modules of BphS‐based CRISPR‐dCas9 transcription system (FACE).^[^ ^37^ ^]^ In contrast, a light‐inducible CRISPR‐dCas9 transcription system based on CRY2/CIB1 (CPTS 2.0)^[^ ^43^ ^]^ has no significant background activity, but it did not elicit biological responses, possibly due to activation strength lower than what was needed to evoke a biological response.

Here, we developed a light‐inducible cell reprogramming (LIRE) system, taking advantage of CRISPR/dCas9 and light‐inducible Tet Repressor (liTetR) based on the plant photoreceptor cryptochrome 2 photolyase homology region (CRY2PHR) and its binding partner: N‐terminal fragment of CIB1 (CIBN).^[^ ^42^ ^]^ With this genetically encoded LIRE system, we can precisely remodel endogenous *Sox2* and *Oct4* genomic loci to convert mouse embryonic fibroblasts (MEFs) into induced pluripotent stem cells (iPSC_LIRE_) under light‐emitting diode (LED)‐based light illumination. Further, using iPSC_LIRE_ cells for blastocyst injection, we generated optogenetic‐controlled chimeric mice from iPSC_LIRE_ cells, which allowed activation of user‐defined endogenous genes in vivo, enabling us to perform any locus‐specific gain‐of‐function studies based on illumination or withdrawal of light. These optogenetic chimera mice will be valuable for facilitating broader applications to uncover unknown gene functions in vivo or for modeling human diseases. This study opens a new direction for reprogramming to pluripotency and cell‐fate determination, and potentially for regenerative medicine, stem cell biology, and disease modeling.

## 2 Results

### 2.1 Design, Optimization, and Characterization of the LIRE System

**Figure 1:**
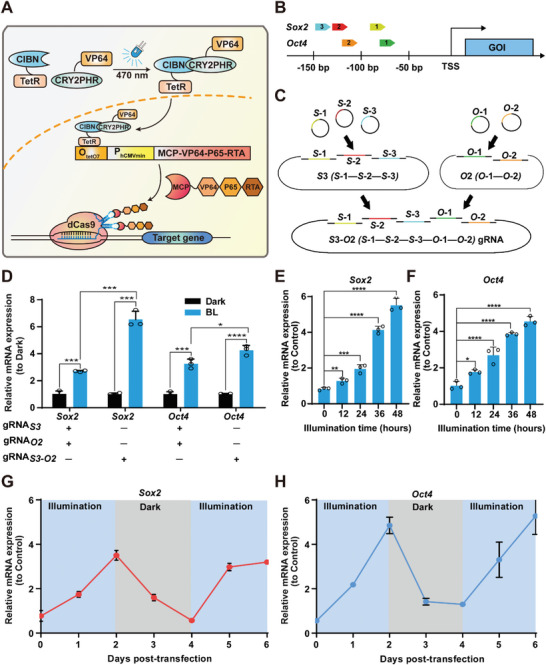
Design, optimization, and characterization of a light‐inducible cell reprogramming (LIRE) system. A) Schematic representation of the LIRE system for controlling transgene expression. In this system, an N‐terminal fragment of CIB1 (CIBN) from *Arabidopsis thaliana* is fused to a Tet Repressor (TetR) DNA‐binding domain to create a fusion light sensor domain (CIBN‐TetR) driven by a constitutive promoter (P_EF1a_). The light‐inducible heterodimerizing protein CRY2PHR is fused to a tetrameric repeat of the minimal *Herpes simplex‐*derived transactivator VP16 (VP64) to create a light‐dependent transactivator (CRY2PHR‐VP64), again driven by a constitutive promoter (P_EF1a_). Upon exposure to blue light, CRY2PHR undergoes a conformational change that enables heterodimerization with CIBN, which induces the expression of the transactivator by assembling MCP and VP64‐p65‐Rta (VPR) from a TetR‐specific inducible promoter P_hCMV*‐1_ (P_hCMV*‐1_, tetO7‐P_hCMVmin_ consisting of an O_TetO7_ sequence upstream to a minimal P_hCMVmin_ promoter) and then further recruited through the MS2 box of the sgRNA‐dCas9 complex to induce downstream gene expression. B) Scheme depicting the sgRNA targeting sites for the promoters of the transcription factor genes *Sox2* and *Oct4* (the transcription start site (TSS) is indicated). C) Schematic representation for the construction of a plasmid for expression of sgRNAs targeting the transcription factors *Sox2* and *Oct4*. D) Optimization of the different configurations of the concatenated (*Sox2* and *Oct4*) sgRNA plasmid. 2 × 10^4^ MEF_liTetR_ stable cells containing the liTetR module were co‐transduced with lentiviral pXS204 (LTR‐P_EF1*α*_‐dCas9‐T2A‐Blasticidin‐LTR), pXS205 (LTR‐P_hCMV*‐1_‐MCP‐VPR‐T2A‐Hygro‐LTR), and pS3 (LTR‐P_U6_‐S84‐P_U6_‐S136‐P_U6_‐S148‐LTR); or pO2 (LTR‐P_U6_‐O71‐P_U6_‐O127‐LTR) or pS3‐O2 (LTR‐P_U6_‐S84‐P_U6_‐S136‐P_U6_‐S148‐P_U6_‐O71‐P_U6_‐O127‐LTR). The cells were subsequently illuminated with pulsed blue light (460 nm, 1.0 mW cm^−2^) for 48 h (1 min on, 5 min off, alternating), and the *Sox2* and *Oct4* mRNA levels were quantified by qPCR (relative to the dark control samples). E,F) Exposure time‐dependent LIRE‐mediated activation of *Sox2* and *Oct4* expression. MEF_liTetR_ stable cells (2 × 10^4^) were co‐transduced with lentiviral pXS204 (LTR‐P_EF1*α*_‐dCas9‐T2A‐Blasticidin‐LTR), pXS205 (LTR‐P_hCMV*‐1_‐MCP‐VPR‐T2A‐Hygro‐LTR), and pS3‐O2 (LTR‐P_U6_‐S84‐P_U6_‐S136‐P_U6_‐S148‐P_U6_‐O71‐P_U6_‐O127‐LTR), and then illuminated with pulsed blue light for different time periods (0–48 h, 1 min on, 5 min off, alternating) and *Sox2* and *Oct4* levels were quantified by qPCR at 48 h after illumination. The data are expressed as the amount of mRNA relative to the control cells transfected with empty vector and kept in the dark. G,H) Reversibility of LIRE‐mediated activation of *Sox2* and *Oct4* transcription. MEF_LIRE_ stable cells were co‐transduced as described in (E) and (F) and subsequently cycled between 2 days of illumination (blue rectangles) and 2 days of dark incubation (grey rectangles) for 6 days. *Sox2* and *Oct4* mRNA levels were quantified by qPCR once a day for 6 days. The data are expressed as the amount of mRNA relative to the control cells transfected with empty vector and kept in the dark. D) Data represent the mean ± SD (*n* = 3 independent experiments) and were analyzed by two‐way ANOVA followed by Tukey's post hoc tests. E,F) Data represent the mean ± SD (*n* = 3 independent experiments) and were analyzed by one‐way ANOVA followed by Tukey's post hoc tests. *p < 0.05, **p < 0.01, ***p < 0.001, ****p < 0.0001.

To precisely remodel endogenous TF loci to initiate reprogramming toward pluripotency, we constructed the LIRE system based on the liTetR and CRISPR/dCas9. In our design, we fused an N‐terminal fragment of CIB1 (CIBN) from *Arabidopsis thaliana* to a Tet Repressor (TetR) DNA‐binding domain to form a hybrid DNA binding protein (CIBN‐TetR). The light‐inducible heterodimerizing protein CRY2PHR was fused to a tetrameric repeat of the minimal *Herpes simplex‐*derived transactivator VP16 (VP64) to form a light‐dependent transactivator (CRY2PHR‐VP64). Under blue light (BL) illumination, CRY2PHR interacts with CIBN, thereby CIBN‐TetR binds to a TetR‐specific inducible promoter P_hCMV*‐1_ (tetO7‐P_hCMVmin_) to initiate gene transcription. Taking advantage of the synergistic activation mediator system,^[^ ^19^ ^]^ which has been demonstrated to confer enhanced efficiency of gene activation using sgRNA bearing MS2 RNA aptamers,^[^ ^25^ ^]^ we first fused the MS2 coat protein (MCP) to VP64‐p65‐Rta (VPR) to form a hybrid transactivator as a linker between our liTetR and the CRISPR‐dCas9 module, which can be recruited to the complex containing sgRNA bearing MS2 aptamers and dCas9 to induce transcriptional activation of the endogenous pluripotency‐related genes for cellular reprogramming upon light illumination (**Figure** **1**A). We next tested the light inducibility of liTetR using an EGFP reporter. Briefly, after co‐electroporating MEFs with a liTetR iteration comprising the fusion light sensor vector (CIBN‐TetR) and the transactivator expression vector (CRY2PHR‐VP64), and the P_hCMV*‐1_‐driven EGFP reporter (tetO7‐P_hCMVmin_‐EGFP), we found that brighter EGFP fluorescence intensity was observed upon BL illumination as compared to the dark control cells (Figure S1, Supporting Information).

To achieve inducible endogenous gene transcription using the LIRE system, we first generated stable MEF cell lines (MEF_liTetR_) harboring the liTetR modules (Figure S2, Supporting Information) which precisely activates endogenous chromatin loci for cellular reprogramming upon light illumination. We selected *Sox2* and *Oct4* as previously identified for triggering cellular reprogramming toward pluripotency^[^ ^11^, ^44^ ^]^ and then optimized the promoter targeting of single gRNAs to the TF genes *Sox2* and *Oct4* in MEF cells (Figure 1B,C). Note that we detected significantly higher *Sox2* and *Oct4* levels when using a single plasmid carrying multiple sgRNAs for *Sox2* and *Oct4* as compared to using individual plasmids for each sgRNA (Figure 1D). Detailed characterization of the LIRE system showed that the extent of *Sox2* and *Oct4* transcriptional induction is exposure time‐dependent (Figure 1E,F), and experiments which oscillated light exposure over 6 days supported dynamic control of endogenous genes *Sox2* and *Oct4* expression (Figure 1G,H), collectively indicating that LIRE system may provide a valuable means to precisely control the dosage of target gene expression.

We next compared the activation efficiency between LIRE system and two existing split‐CPTS 2.0 and CPTS system in MEF cells. The LIRE system efficiently activated the endogenous *Sox2* and *Oct4* gene transcription upon light illumination compared to CPTS 2.0 and significantly decreased leakiness in the dark compared to split‐CPTS 2.0. These results demonstrated our LIRE system showed high light/dark contrasts and no obvious leak activity in the dark (*Sox2*: 8.7‐fold induction; *Oct4*: 7.1‐fold induction) compared to either split‐CPTS 2.0 (*Sox2*: 4.62‐fold induction; *Oct4*: 3.79‐fold induction) or CPTS 2.0 (*Sox2*: 4.66‐fold induction; *Oct4*: 5.73‐fold induction) (Figure S3, Supporting Information).

### 2.2 Reprogramming of Fibroblasts into iPSCs Using the LIRE System

To determine whether the LIRE system with sgRNAs targeting *Sox2* and *Oct4* could optogenetically activate the pluripotency genes, we transduced MEF_liTetR_ cells with dCas9 and the sgRNAs targeting *Sox2* and *Oct4* (Figure S4A, Supporting Information) and used qPCR to assess *Sox2* and *Oct4* levels after 4 days of light illumination. *Sox2* and *Oct4* were transcriptionally activated upon illumination (Figure S4B, Supporting Information), and subsequent immunofluorescence (IF) imaging analysis confirmed the BL‐induced increases in the Sox2 and Oct4 protein levels (Figure S4C, Supporting Information).

**Figure 2:**
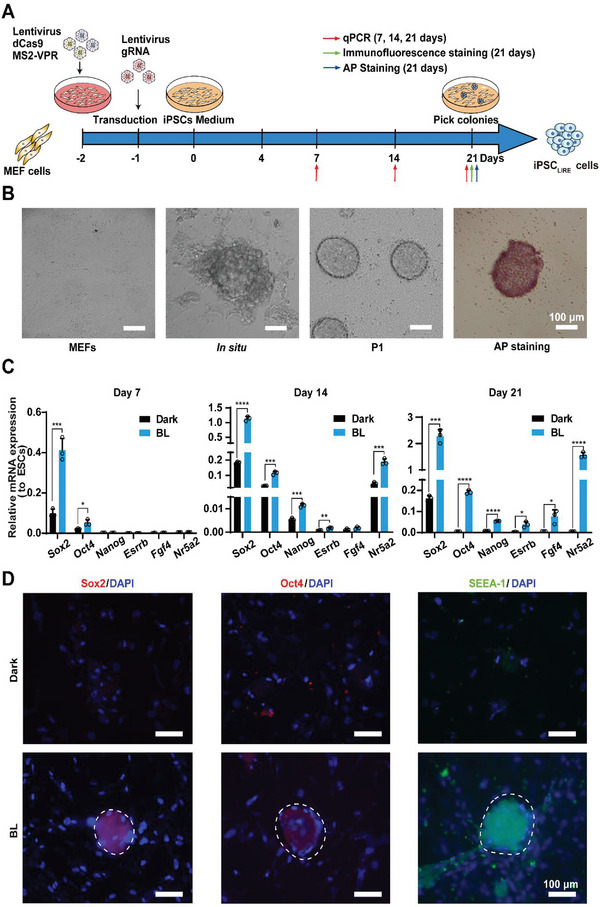
Light inducible reprogramming MEF cells to iPSC_LIRE_ cells using LIRE system. A) Schematic showing the experimental procedure and the time schedule used for reprogramming MEFs to iPSC_LIRE_ cells. B) Representative microscopy images of MEFs on day 0 and iPSC‐like colonies (iPSC_LIRE_) in situ and at passage 1, as well as AP‐positive iPSC_LIRE_ from light‐illuminated cells. Scale bar = 100 µm. C) Temporal expression patterns of genes by qPCR analysis during light inducible reprogramming toward pluripotency. MEF_liTetR_ stable cells (2 × 10^4^) were transduced with lentiviral pXS204 (LTR‐P_EF1*α*_‐dCas9‐T2A‐Blasticidin‐LTR), pXS205 (LTR‐P_hCMV*‐1_‐MCP‐VPR‐T2A‐Hygro‐LTR), and pS3‐O2 (LTR‐P_U6_‐S84‐P_U6_‐S136‐P_U6_‐S148‐P_U6_‐O71‐P_U6_‐O127‐LTR), and then illuminated with pulsed blue light (460 nm, 1.0 mW cm^−2^) for 7 days (0.5 min on, 5 min off, alternating). The levels of the mRNA for pluripotency genes (*Sox2*, *Oct4*, *Nanog*, *Esrrb*, *Nr5a2*, and *Fgf4*) were quantified by qPCR on days 7, 14, and 21. The data are expressed as the amount of mRNA relative to the positive controls (ESCs). D) Representative fluorescence microscopy images of pluripotency markers including Sox2 (Red), Oct4 (Red), and SSEA‐1 (Green) in iPSC_LIRE_ colonies derived from light‐inducible MEF_liTetR_cells on day 21. Blue indicates DAPI staining of nuclei. The images represent typical results from three independent measurements. Data in (C) represent the mean ± SD (*n* = 3 independent experiments) and were calculated by Student's *t*‐test. *p < 0.05, **p < 0.01, ***p < 0.001, ****p < 0.0001.

We next explored whether the LIRE‐system‐induced transcriptional activation of *Sox2* and *Oct4* had functional consequences, specifically examining the potential reprogramming of MEFs into iPSCs upon illumination by LED‐based light. After transduction of lentiviral dCas9 and the plasmid with sgRNAs for *Sox2* and *Oct4* activation, the MEF medium was changed to reprogramming medium.^[^ ^44^ ^]^ qPCR analysis of known pluripotency marker genes was conducted at various times during the putative reprogramming process to determine the degree to which the MEFs were reprogrammed into iPSCs (**Figure** **2**A–C). The pluripotency markers *Sox2*, *Oct4*, *Nanog*, *Esrrb*, *Fgf4*, and *Nr5a2* were gradually upregulated with time, and were highly expressed on day 21 in the illuminated group cells as compared to cells in the dark (Figure 2C) and these cells stained positive for the pluripotency markers Sox2, Oct4, and SSEA‐1 (Figure 2D and Figure S5, Supporting Information) and alkaline phosphatase (AP) (Figure 2B). Meanwhile, morphological changes were observed from day 7, and iPSC‐like colonies were visible on day 14 in the illuminated group, while no colonies were detected on plates that were not exposed to light or treated for less than 14 days (Figure S6, Supporting Information). Moreover, the picked iPSCs colonies were exposed to BL to determine whether these cells carried LIRE system integrations. BL illumination significantly induced Gaussia Luciferase (GLuc) expression compared to control cells incubated in the dark after transduction of a Gluc reporter (Figure S7, Supporting Information), and also significantly induced expression of the endogenous gene *Sox2* and *Oct4* compared to control cells kept in the dark after transduction of lentiviral dCas9 and sgRNAs targeting *Sox2* and *Oct4* (Figure S8, Supporting Information). These data demonstrate that iPSC_LIRE_ cells achieve the pluripotent signatures with light‐responsive ability.

**Figure 3:**
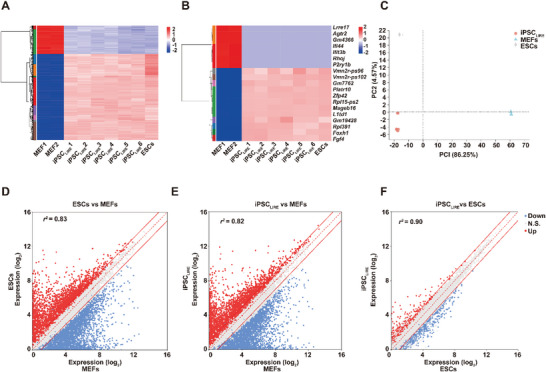
Transcriptional analysis of iPSC_LIRE_. A) Heatmap of unsupervised hierarchical clustering based on expression of 500 significantly fluctuated genes in MEFs, different iPSC_LIRE_ colonies, and ESCs (positive control). The abscissa is the sample, and the ordinate is the genes. Different colors indicate different gene expression levels, and the color ranges from blue to white to red, indicating low to high expression levels. B) Clustering of MEF, different iPSC_LIRE_ colonies, and the positive control ESCs based on expression of 20 significantly fluctuated genes. C) Principal component analysis (PCA) of MEFs, iPSC_LIRE_ colonies, and the positive control ESCs based on expression of the differentially regulated genes (86.25% of the variation in PC1 and 4.57% of the variance in PC2). D–F) Scatter plots comparing global gene expression profiles between MEFs and ESCs, MEFs and iPSC_LIRE_, and ESCs and iPSC_LIRE_. Correlation was assessed using Pearson's correlation coefficient (*r*). The central red dashed line represents equal gene expression and the outer red solid lines indicate twofold different expression.

To further compare the LIRE system‐induced iPSC_LIRE_ cells with embryonic stem cells (ESCs), we examined the global transcriptome of MEF cells, iPSC_LIRE_ cells, and ESCs. Hierarchical clustering analysis showed iPSC_LIRE_ cells were closely clustered with ESCs and were distinct from that of MEF cells (**Figure** **3**A and Figure S9A,B, Supporting Information). Moreover, iPSC_LIRE_ cells expressed numerous genes primarily associated with pluripotency at levels similar, but not identical, to those of ESCs (Figure 3B). Principal component analysis of the gene expression data also showed two clearly distinct clusters among these cells (Figure 3C). Moreover, scatter plot analysis showed a tight correlation in gene expression between iPSC_LIRE_ cells and ESCs (Figure 3D–F) indicating almost no difference between iPSC_LIRE_ cells and ESCs in global gene expression profiles. Taken together, these results indicated that iPSC_LIRE_ cells closely resemble ESCs in global gene expression profiles.

### 2.3 Pluripotency Characteristics of iPSC_LIRE_ Cells Equipped with LIRE System

**Figure 4:**
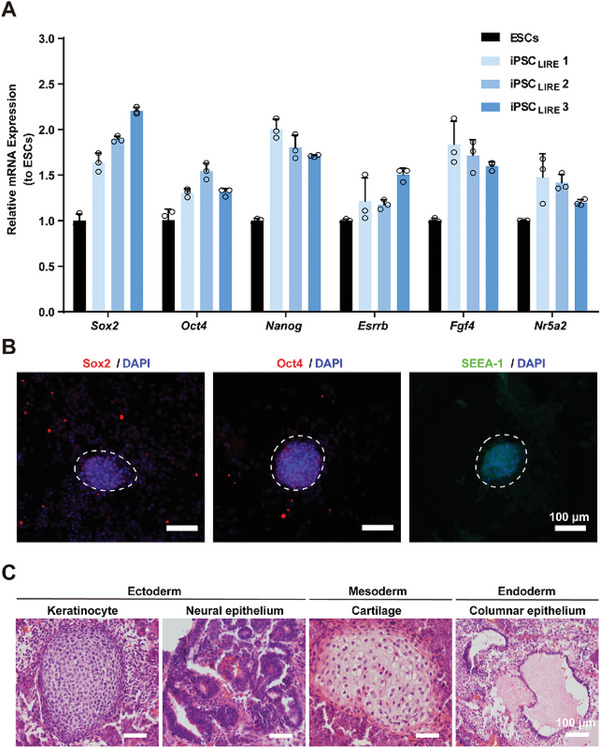
Pluripotency characteristics of light‐inducible iPSC_LIRE_. A) qPCR analysis of pluripotency marker genes (*Nanog*, *Esrrb*, *Nr5a2*, and *Fgf4*) in three separate light‐inducible iPSCs_LIRE_ lines derived from MEFs under BL illumination. The data are expressed as the amount of mRNA relative to the positive controls (ESCs). B) Representative fluorescence microscopy images of pluripotency markers in the iPSC_LIRE_. Blue indicates DAPI staining of nuclei; Red indicates Sox2 and Oct4; Green indicates SSEA‐1. The images represent typical results from two independent measurements. Scale bar = 100 µm. C) Representative microscopy images of H&E staining of teratomas from iPSC_LIRE_ containing three germ lineages including keratinocyte with keratin and neural epithelium (ectoderm), cartilage (mesoderm), and columnar epithelium (endoderm). 3 × 10^6^ iPSC_LIRE_ were subcutaneously injected in each mouse. After 4 weeks, the tissue sections from teratoma stained with hematoxylin and eosin. The images represent typical results from two independent measurements. Scale bar = 100 µm. Data in (A) represent the means ± SD; *n* = 3 independent experiments.

We further examined whether the iPSC_LIRE_ cells were authentically pluripotent. The iPSC_LIRE_ colonies were selected and expanded into iPSCs lines, each showing typical mouse ES‐like domed colony morphology. qPCR showed that these iPSC_LIRE_ cells expressed pluripotency markers *Sox2*, *Oct4*, *Nanog*, *Esrrb*, *Fgf4*, and *Nr5a2* at levels comparable to mouse ESCs (**Figure** **4**A). Immunofluorescent staining results further confirmed expression of pluripotency markers in these iPSC_LIRE_ cells, including Sox2, Oct4, and SSEA‐1 (Figure 4B and Figure S10, Supporting Information). In addition, these cells can be passaged for more than 20 passages maintaining iPSC‐like colony morphology (Figure S11A, Supporting Information) and optogenetic control of gene expression (Figure S11B, Supporting Information). Karyotype analysis revealed that the iPSC_LIRE_ cell exhibited a normal karyotype of 40XY (Figure S12, Supporting Information).

We additionally conducted teratoma assays—a classic experiment for evaluating pluripotency and multidifferentiation of ES cells or iPSCs.^[^ ^45^ ^]^ After subcutaneously injecting iPSC_LIRE_ cells into immunocompromised, non‐obese diabetic‐severe combined immunodeficient (NOD‐SCID) mice (Figure S13A, Supporting Information), teratoma formation was easily observed after 4 weeks (Figure S13B, Supporting Information). Moreover, histological analysis of teratoma sections revealed three embryonic germ layers (Figure 4C), including typical keratinocyte‐like and neural epithelium‐like tissues (ectodermal lineage), cartilage‐like tissues (mesodermal lineage), and columnar epithelium tissues (endodermal‐lineage), supporting both the pluripotency and multidifferentiation capacity of the iPSC_LIRE_ cells in vivo. We subsequently established fibroblast‐like cells from iPSC_LIRE_ cell‐derived teratomas in explant cultures (Figure S14A, Supporting Information) and found that the light illumination significantly induced activation of a Gluc reporter in these fibroblasts when compared to control cells incubated in the dark (Figure S14B, Supporting Information), indicating that these fibroblasts from teratomas still had capability of light‐inducible gene expression using BL as an inducer modality.

The ability of iPSC to differentiate into all cell types is the basis for their potential in regenerative medicine. These iPSC_LIRE_ cells can in theory be used for lineage‐directed differentiation studies under light illumination. To demonstrate that light illumination can trigger cell differentiation based on activation of user‐defined endogenous TFs of interest, we created lentiviral vectors containing two sgRNAs targeting *Neurog2*, a TF that functions in the induction of neuronal differentiation from iPSCs^[^ ^46^, ^47^, ^48^ ^]^ (Figure S15A,B, Supporting Information) or sgRNAs targeting myogenic differentiation genes: mouse laminin subunit alpha 1 (*Lama1*) and follistatin (*Fst*).^[^ ^49^ ^]^ qPCR analysis showed that *Neurog2, Lama1*, or *Fst* levels were significantly increased in illuminated cells compared to dark control cells (Figure S15C,D, Supporting Information). During differentiation of the iPSC toward neuronal cells, typical neural‐lineage markers including *Pax6*, *N‐cadherin*, *Tuj1*, and *Nestin* were strongly induced in the illuminated cells compared to dark control cells on day 8 (Figure S15E, Supporting Information). However, low levels of *Sox2* and *Oct4* expression were detected under light illumination (Figure S15F, Supporting Information). In addition, immunofluorescence staining results revealed these cells were stained positively for the neuronal markers neurofilament 200 and beta III tubulin (Tuj1) (Figure S16, Supporting Information) and calcium ion imaging showed that the differentiated neuronal cells displayed significant changes in the intracellular calcium ion (Video S1, Supporting Information). Collectively, a simple introduction of sgRNAs in iPSC_LIRE_ cells can temporally activate lineage‐specific TFs and thereby affect cell fate transitions under light illumination.

### 2.4 Generation of Optogenetic Mice with LIRE System

**Figure 5:**
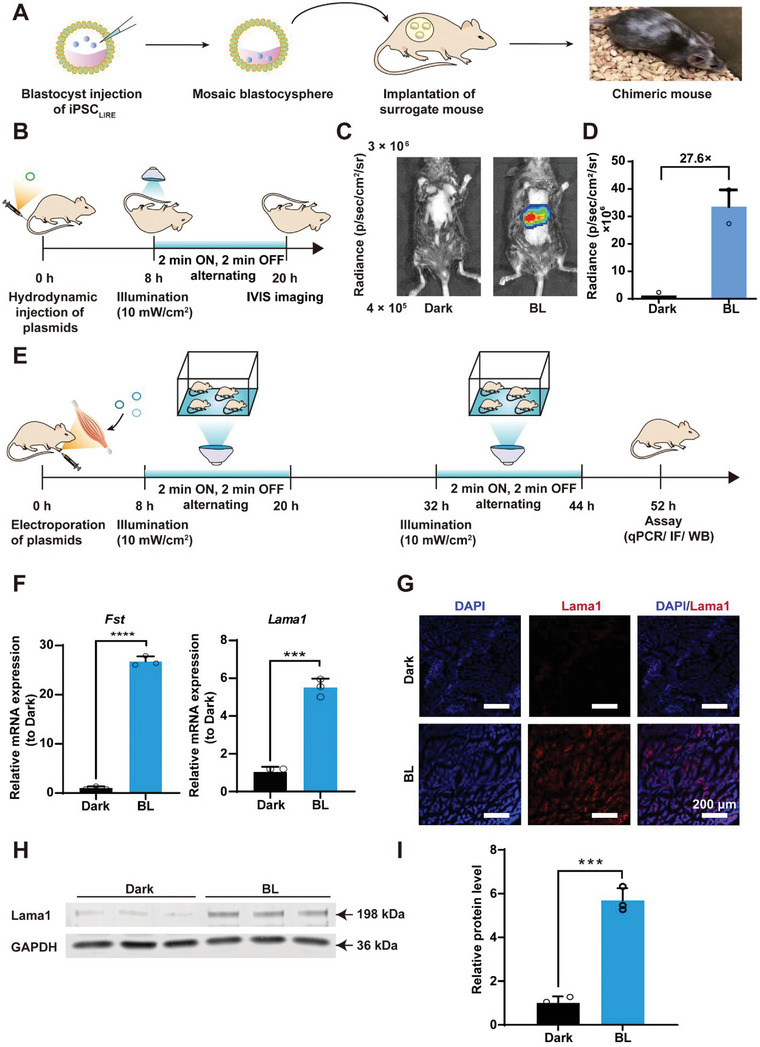
Characterization of the optogenetic chimeric mice generated from iPSC_LIRE_ cells. A) Schematic showing the procedure for the generation of optogenetic chimeric mice. B) Schematic representation of the experimental procedure and the time schedule for LIRE‐mediated activation of exogenous genes in mice. The optogenetic chimeric mice were transiently hydrodynamically injected (tail vein) with a tetO7‐based luciferase reporter. At 8 h after injection, the mice were either illuminated with blue light (460 nm, 10 mW cm^−2^) for 12 h (2 min on, 2 min off, alternating) or kept in the dark. Bioluminescence imaging was performed 20 h after hydrodynamic injection. C) Bioluminescence IVIS image of the luciferase reporter gene activation in optogenetic chimeric mice. D) Quantification of the bioluminescence activity shown in (C). E) Schematic illustrating the experimental procedure and the time schedule used for LIRE‐mediated activation of the endogenous genes in mouse muscles. The tibialis anterior (TA) and tibialis posterior (TP) muscles of the mice were electroporated with 40 µg of BL‐inducible transactivator vector pXS205 (LTR‐P_hCMV*‐1_‐MCP‐VPR‐T2A‐Hygro‐LTR) and the concatenated dCas9 and sgRNA1 vector pGY174 (P_U6_‐sgRNA1[*Lama1*]‐pA‐P_U6_‐sgRNA2[*Lama1*]‐pA‐P_hCMV_‐dCas9‐pA) or pGY177 (P_U6_‐sgRNA1[*Fst*]‐pA‐P_U6_‐sgRNA2[*Fst*]‐pA‐P_hCMV_‐dCas9‐pA), and the mice were either exposed to blue light (460 nm, 10 mW cm^−2^) for 8 h/day for 2 days (2 min on, 2 min off, alternating) or kept in the dark. Muscle tissues were harvested at 3 days after electroporation and assessed with qPCR, immunoblotting, and cryosection staining. F) qPCR analysis of light‐induced activation of *Lama1* and *Fst* transcription in leg muscles. G) Representative fluorescence microscopy images of Lama1 immunostaining in a TP muscle section. Blue indicates DAPI staining of nuclei; Red indicates Lama1. The images represent typical results from two independent measurements. Scale bar = 100 µm. H) Western blot analysis of light‐induced activation of the endogenous Lama1 protein expression in the leg muscles. Western blotting images were acquired from two independent experiments. I) Quantification of the endogenous protein levels of Lama1 shown in (H). D,F,I) Data represent the mean ± SEM, *n* = 4 mice, and were analyzed by Student's *t*‐test. ***p < 0.001, ****p < 0.0001.

The most stringent criterion for assessing iPSCs’ pluripotency is generation of chimeric animals.^[^ ^50^ ^]^ After confirming pluripotency of iPSC_LIRE_ cells with light‐responsive ability, we determined whether the iPSC_LIRE_ cells could produce light‐inducible chimera mice. We performed a blastocyst injection assay to test the stringent pluripotency of iPSC_LIRE_ cells. After iPSC_LIRE_ cells were injected into the inner cell masses of C57BL/6J mouse blastocysts, live‐born chimeric mice containing the LIRE system were generated (**Figure** **5**A and Figure S17, Supporting Information), and the chimeric rate was 30.8% (Table S5, Supporting Information, 4 out of 13). These data confirm their pluripotency in normal development.

We subsequently tested whether the chimeric mice produced by the donor iPSC_LIRE_ cells enable optogenetic regulation of gene expression in vivo. We used hydrodynamic tail vein injection to transiently transfect the optogenetic chimeric mice with a P_hCMV*‐1_‐driven luciferase reporter plasmid. 8 h after injection, the mice were externally illuminated with BL (460 nm, 10 mW cm^−2^) for 12 h (2 min on, 2 min off) and luciferase production was quantified by bioluminescence (Figure 5B). Compared to the dark control animals, the mice that received illumination exhibited a significant increase in luciferase activity in the liver (a 27.6‐fold increase in bioluminescence radiance) (Figure 5C,D). This result demonstrates that optogenetic chimera mice can respond to external light induction.

We then explored in vivo activation of user‐defined endogenous genes in the optogenetic chimera mice based on non‐invasive external illumination using an LED light source. We targeted the mouse laminin subunit alpha 1 (*Lama1*) and follistatin (*Fst*) genes because increases in *Lama1* and *Fst* levels have been linked to increased muscle cell quantity and function.^[^ ^51^ ^]^ The tibialis anterior and tibialis posterior muscles of 6‐week‐old optogenetic chimera mice were electroporated with a total of 40 µg of the plasmids for CRISPR/dCas9 or two kinds of sgRNAs that specifically target the promoter regions of the *Lama1* or *Fst* loci, followed by illumination with BL (460 nm, 10 mW cm^−2^) for 12 h (2 min on, 2 min off) each day for 2 days (Figure 5E). qPCR analyses showed that *Lama1*and *Fst* were upregulated, while *Sox2* and *Oct4* expression were not obviously detected in the chimeric mice under light illumination (Figure 5F and Figure S18, Supporting Information), which indicated that light exposure may not induce unwanted reprograming in optogenetic chimeric mice. In addition, immunocytochemistry and western blot analyses showed that light‐induced an increase in the Lama1 protein level in isolated muscle tissues; no signal for Lama1 was detected in muscle tissues of dark control mice (Figure 5G–I). Finally, fibroblasts were isolated from the optogenetic chimera mice to determine whether the integrated LIRE system retained competence to mediate light‐inducible gene expression after differentiation in the developing embryo. As expected, illumination of the fibroblasts generated from the optogenetic chimera mice (Figure S19A, Supporting Information) resulted in significant activation of an electroporation‐delivered EGFP reporter when compared to control dark cells (Figure S19B, Supporting Information). Collectively, these results demonstrate that optogenetic chimera mice can respond to light induction to activate the transcription of user‐defined endogenous genes of interest, thus providing an optogenetic mouse system that can enable in vivo studies of gene function and the genetics of disease.

## 3 Discussion

Current cellular reprogramming technologies are mainly dependent on overexpressive ectopic TFs to trigger multiplexing gene regulatory networks leading to cell transitions.^[^ ^52^, ^53^ ^]^ However, preset overexpression may not result in the desired outcome because there is no general guarantee that a given network's dynamics will allow transitions to the desired target state. An ideal cellular reprogramming system could provide fine‐tunable and robust transcriptional control of the core TFs determining cell‐fate decision and lineage commitment. However, rapid and traceless changes of gene expression patterns are difficult to address using classical chemically regulated gene expression systems due to their poor temporal and highly invasive characteristics. Moreover, chemical inducers are difficult to be removed and some chemicals such as rapamycin may also induce side effects by perturbing the endogenous mTOR pathway.^[^ ^54^, ^55^ ^]^ Light processing has emerged as an innovative and powerful approach to modulate cell behavior because of the remote, traceless, and non‐invasive properties.^[^ ^30^, ^37^, ^43^, ^56^, ^57^, ^58^, ^59^ ^]^ In the present study, we developed a LIRE system based on a light‐responsive optogenetic device and the CRISPR/dCas9 system that can efficiently control the transcription of multiplexed endogenous TFs using sgRNA molecules in a light‐dependent manner. This type of approach allows for targeting gene regulatory networks with precise regulation of endogenous TFs for cell lineage conversion.

There is now extensive support for the ideas that *Sox2* and *Oct4* represent the core regulatory circuitry of pluripotency.^[^ ^60^, ^61^ ^]^ We demonstrate an alternative approach for iPSCs generation using the LIRE system based on the optogenetic control of endogenous *Sox2* and *Oct4* with introduction of a combination of all‐in‐one multiplexed gRNA expression vector. Notably, the unwanted induction of Sox2 and Oct4 was not observed in the generated optogenetic iPSCs and chimeric mice when exposed to light without additional lentiviral delivery of dCas9, MCP‐VPR, and sgRNAs. This is probably due to the low level of sgRNA transcribed from the U6 promoter^[^ ^62^ ^]^ and more susceptibility to nucleolytic degradation,^[^ ^63^ ^]^ as well as the structure change of the integrated sgRNA that might affect its binding to dCas9.^[^ ^64^ ^]^

Despite generation of optogenetic iPSCs, there is still room for improvement of our LIRE system. Using LIRE system, we will further investigate the expression levels of the two endogenous TFs *Sox2* and *Oct4* at different time points and analyze the timing of the reprogramming process which allows for more efficient reprogramming. Moreover, advancement of delivery strategies, including adeno‐associated viruses^[^ ^65^ ^]^ or nonviral vectors including the lipid nanoparticles,^[^ ^66^ ^]^ may help increase the efficiency of delivery for our system avoiding drawbacks of the used lentiviral vectors such as random insertion.

In addition, we demonstrate that light illumination enabled activation of the endogenous TFs driving neural or muscle cells differentiation with the help of sgRNAs targeting specific TFs, which supported iPSC_LIRE_ cell differentiation into neural or muscle cells. In theory, our generated iPSC_LIRE_ cells could differentiate into different types of cells after introduction of sgRNAs targeting corresponding TFs under LED light. In the future, this system may be combined with other light‐inducible strategies for controlling tissue development. Therefore, the LIRE system developed here offers an accurate and efficient platform for inducing pluripotent cells and cell fate change.

Importantly, we demonstrate that iPSC_LIRE_ cells can be used to produce optogenetic chimera mice, and we confirmed that these mice respond well to external LED light illumination, which provide in vivo light inducible gene regulation system for major unmet biomedical research. More attractively, our optogenetic chimera mice can enable traceless and non‐invasive gene manipulations in vivo which should facilitate discoveries about embryonic development and/or stem cell differentiation and help to dissect the regulatory networks orchestrating vertebrate development and engineer superior tissue constructs for basic research and regenerative medicine applications, based on simple intraperitoneal injection of dCas9/gRNAs and LED light illumination. Further, by performing crosses of the optogenetic chimera mice with existing genetically engineered mouse models, researchers can model complex human diseases to dissect endogenous gene function in vivo for therapeutic benefit in cell‐transplantation therapy.

Collectively, our LIRE system represents a convenient, traceless, and non‐invasive reprogramming strategy for inducing pluripotent cells by optogenetic control of targeting loci of the endogenous TFs. We generated iPSCs responsive to light induction and obtained optogenetic chimeric mice, which provides a valuable tool for gene function studies of gene regulatory networks and in vivo genetic screens. We expect that our LIRE system will pave the way for broader applications including basic research for dissecting spatiotemporal molecular mechanisms and potential therapeutic applications in cell therapies and regenerative medicine.

## 4 Experimental Section

### Materials and Reagents

Materials, reagents, and antibodies used in this study are provided in Table S1, Supporting Information.

### Cloning and Plasmid Construction

Plasmids and primers used in this study are provided in Table S2, Supporting Information, and the sequences were confirmed by DNA sequencing (Genewiz Inc., Suzhou, China). The detailed DNA sequence information of the most important genetic modules used in this study is provided in the Supporting Information. Some plasmids were constructed by ClonExpress MultiS One Step Cloning Kit (Vazyme Inc.; Nanjing, China; Catalog no. C113‐01) according to the manufacturer's instructions.

### Cell Culture

Human embryonic kidney cells (HEK‐293T, ATCC: CRL‐11268) were cultured at 37 °C in a humidified atmosphere containing 5% CO_2_ in Dulbecco′s Modified Eagle Medium (DMEM, Gibco; NY, USA; Catalog no. 31600‐083) containing 10% v/v fetal bovine serum (FBS, Gibco; Catalog no. 10270‐106) and 1% v/v penicillin/streptomycin solution (Sangon Biotech; Shanghai, China; Catalog no. B540732). MEFs were prepared from the E13.5 embryos of CD‐1 mouse and cultured in DMEM supplemented with 10% v/v FBS (Gibco; Catalog no. 16000‐044), non‐essential amino acid (NEAA, Gibco; Catalog no. 11140‐050), and 1% v/v penicillin/streptomycin solution.

All the iPSCs and ESCs were cultured in 0.1% w/v gelatin‐coated dishes in Glasgow Minimum Essential Medium (Gibco; Catalog no. 11710‐035) with 15% FBS (Gibco; Catalog no. 16000‐044), 1% v/v penicillin/streptomycin solution, 1% v/v GlutaMAX (Gibco; Catalog no. 35050‐061), 1% v/v NEAA, 0.1 × 10^−3^ m *β*‐mercaptoethanol (Sigma‐Aldrich; St. Louis, MO, USA; Catalog no. M3148), 1000 U mL^−1^ recombinant mouse leukemia inhibitory factor (LIF, Millipore; Boston, MA, USA; Catalog no. ESG1107), 3 µm CHIR99021 (Seleckchem; Houston, Texas, USA; Catalog no. S2924), and 1 µm PD0325901 (Seleckchem; Catalog no. S1036). All cells were regularly tested for the absence of *Mycoplasma* and bacterial contamination.

### Isolation of MEF Cells

E13.5 embryos were used for MEF isolation. After the embryo recovery, the head, tail, and internal organs were removed. The remaining bodies of the embryos were finely minced with the blade and digested in 0.25% Trypsin‐EDTA for 10 min. Cells were then collected by centrifugation at 800 × *g* for 10 min and plated onto 75 cm^2^ cell culture flasks. MEF cells were used in early passages (before passage 5) for all tests.

### Generation of Stable Cell Lines

The MEF_liTetR_ stable cells, transgenic for the constitutive light‐inducible system were constructed by co‐transducing MEF cells with a transactivator expression lentiviral vector pXS206 (LTR‐P_EF1*α*_‐CRY2PHR‐VP64‐T2A‐Puromycin‐LTR) and a light sensor vector pXS207 (LTR‐P_EF1*α*_‐TetR‐CIBN‐T2A‐Zeocin‐LTR). After selection with 1 µg mL^−1^ puromycin (Thermo Fisher Scientific; Waltham, MA, USA; Catalog no. A1113803) and 100 µg mL^−1^ zeocin (Thermo Fisher Scientific; Catalog no. R25001) for 2 weeks, the surviving population was selected and picked for further cultivation. All stable cells were regularly tested for the absence of *mycoplasma* and bacterial contamination.

### Lentivirus Preparation and Transduction

HEK‐293T cells (5 × 10^6^) were plated into a 15 cm dish and cultured for 16 h before transfection and were subsequently co‐transfected with a plasmid encoding the desired gene of interests (15 µg), lentiviral package plasmid (psPAX2, 15 µg, Addgene; Catalog no. 12260), and a plasmid encoding for VSV‐G pseudotyping coat protein (pMD2.G, 7.5 µg, Addgene; Catalog no. 12259) using an optimized polyethyleneimine (PEI)‐based protocol with 1.5 mL of a 3:1 PEI:DNA mixture (w/w) (PEI, MW 40000, stock solution 1 mg mL^−1^ in ddH_2_O; Polysciences; Catalog no. 24765). After 6 h, media were changed. Supernatant containing the virus was harvested at 48 h post‐transfection and filtered through a 0.45 µm syringe filter (Pall Corporation; NY, USA; Catalog no. 4654) to remove cell debris. Then the filtered supernatants were concentrated by ultracentrifugation at 25 000 × *g* for 2 h at 4 °C using a Beckman Avanti J‐26 XPI centrifuge with a JA‐25.50 rotor (Beckman Coulter, Inc.; CA, USA), and virus pellets were re‐suspended with 300 µL phosphate buffered saline (PBS) for immediate use or stored at −80 °C before use.

For transduction, MEF cells were transduced with the corresponding lentiviral vectors in the presence of 10 µg mL^−1^ polybrene (Sigma‐Aldrich; Catalog no. H9268) for 16 h. Two rounds of virus transduction were performed as the first round for lentiviral encoding dCas9, MCP‐VPR, and the second round for lentiviral encoding sgRNAs. Media were changed after each transduction.

### Titration of Lentiviral Vectors

Lentiviral vector titration was determined using quantitative real‐time PCR (qRT‐PCR) methods. A total of 5 × 10^4^ MEFs per well were seeded into a 6‐well culture plate 1 day before transduction. Lentiviral transduction was performed with serial dilutions of virus stocks in a total of 0.5 mL medium containing 10 µg mL^−1^ polybrene. After 24 h at 37 °C, 2 mL medium was added and the cell plates were incubated at 37 °C for additional 48 h. The treated cells were trypsinized using 0.25% trypsin‐EDTA solution for 3 min at 37 °C and collected for isolating genomic DNA using a TIANamp Genomic DNA Kit (Tiangen Biotech [Beijing] Co., Ltd.; China; Catalog no. DP304) according to the manufacturer's instructions. The number of integrated lentiviruses was determined by qRT‐PCR. qRT‐PCR primers were designed to amplify the gene expression of *Sox2, Oct4*, and the LIRE system components in the genome. Plasmids containing the targets were used to generate standard curve which was plotted with threshold cycle (Ct) values of samples on *y*‐axis and the plasmid copy of a serial of plasmid dilutions (10, 1, 0.1, and 0.01 pg) on the *x*‐axis. The copy numbers of *Sox2, Oct4*, and the LIRE system components in around 50 ng of genomic DNA were calculated based on the standard curves.

### Light‐Controlled Gene Activation in MEF Cells

For exogenous gene activation experiments, 1 × 10^5^ cells were nucleofected with 100 ng of the fusion light sensor vector pXS172, 100 ng of the transactivator expression vector pXS173, and 100 ng of the exogenous P_hCMVmin*_‐driven EGFP reporter pXS170 (P_hCMVmin*_‐EGFP‐pA; P_hCMVmin*_, tetO7‐P_hCMVmin_) using a P4 Primary Cell 4D‐Nucleofector X Kit S (Lonza, Basel, Switzerland; Catalog no. V4XP‐4032) and the CZ‐167 program. After 24 h transduction, cells were illuminated for 2 days (1 min on, 5 min off, alternating) at light intensities of 1.0 mW cm^−2^ using a custom‐designed 4 × 6 blue‐light LED array (460 nm, Epistar Inc., Taiwan, China). The fluorescence of EGFP was measured by a fluorescence microscopy.

For endogenous gene activation in MEF cells, MEFs (1 × 10^5^) were co‐electroporated with the LIRE system (pXS206, pXS207, pXS204 [LTR‐P_EF1*α*_‐dCas9‐T2A‐Blasticidin‐LTR], pXS205 [LTR‐P_hCMV*‐1_‐MCP‐VPR‐T2A‐Hygro‐LTR]) or CPTS 2.0 (pCMV‐NLSdCas9‐NLS, pCMV‐NLSx3‐MS2‐CIB1, pCMV‐NLSx3‐CRY2‐p65‐HSF1) or Split‐CPTS2.0 (pCMV‐NES‐dCas9N‐pMag‐NES, pCMV‐nMagHigh1‐dCas9C‐NLS‐VP64, pCMV‐NLS‐MS2ΔFG‐NLSp65‐HSF1), and the gRNA targeting the *Sox2* and *Oct4* locus (pS3‐O2) at a 1:1:1:1:1 ratio, and then illuminated with pulsing light (460 nm, 1.0 mW cm^−2^) for 2 days (1 min on, 5 min off, alternating).

For endogenous gene activation in MEF cells stably integrated with liTetR module. MEF_liTetR_ cells were co‐transduced with lentivirus containing pXS204 (LTR‐P_EF1*α*_‐dCas9‐T2A‐Blasticidin‐LTR), pXS205 (LTR‐P_CMV_‐MS2‐VPR‐T2A‐Hygro‐LTR), and corresponding sgRNAs pS3 (LTR‐P_U6_‐S84‐P_U6_‐S136‐P_U6_‐S148‐LTR) or pO2 (LTR‐P_U6_‐O71‐P_U6_‐O127‐LTR) or pS3‐O2 (LTR‐P_U6_‐S84‐P_U6_‐S136‐P_U6_‐S148‐P_U6_‐O71‐P_U6_‐O127‐LTR). After 24 h transduction, the culture plate was placed below a custom‐designed 4 × 6 LED array (460 nm; each LED was centered above a single well; Epistar Inc.) and illuminated for different time periods (1 min on, 5 min off, alternating for 12, 24, 36, and 48 h) at light intensities of 1.0 mW cm^−2^. The cells were harvested and total RNA was extracted for qRT‐PCR analysis.

### Cell Reprogramming and iPSC_LIRE_ Derivation

The MEF_LIRE_ stable cells (5 × 10^4^) were plated per well of a gelatin‐coated six‐well plate at 24 h before transduction. After transducing with lentiviral pXS204 (LTR‐P_EF1𝛼_‐dCas9‐LTR), pXS205 (LTR‐P_hCMV*‐1_‐MCP‐VPR‐LTR), and pS3‐O2 (LTR‐P_U6_‐S84‐P_U6_‐S136‐P_U6_‐S148‐P_U6_‐O71‐P_U6_‐128‐LTR), cells were allowed to recover in MEF medium for 24 h. To start cell reprogramming, cultures were switched to the reprogramming medium (ESCs medium), followed by illumination with light (460 nm, 1.0 mW cm^−2^) for 10 days (1 min on, 5 min off, alternating). Cell culture medium was changed every other day until iPSC‐like colonies appeared. After colony formation, light was shut off and cell culture medium was changed every day.

For iPSCs derivation, single colonies were picked up and digested in 0.25% trypsin‐EDTA for 3 min for single‐cell suspension. Cells were then seeded on a six‐well plate in ESCs medium, and these cells were considered as P0 iPSC_LIRE_ cells.

### Gaussia Luciferase Assay

Supernatants were collected from cell culture medium and 20 µL supernatants were added to 20 µL coelenterazine at a working concentration of 1.25 µg mL^−1^. Luminescence measurements were performed on the Synergy H1 hybrid multi‐mode microplate reader (BioTek Instruments, Inc.; TA, USA) and luminescence intensity was expressed as relative light units.

### EGFP Fluorescence Imaging

Fluorescence image of EGFP expression cells were obtained using an inverted fluorescence microscope (Olympus IX71, TH4‐200; Olympus Corporation; Tokyo, Japan) equipped with an Olympus digital camera (Olympus DP71, Olympus Corporation); a 495/535‐nm (B/G/R) excitation/emission filter set and images were acquired with 480 nm excitation and 535 nm emission filters for EGFP signal.

### qPCR Analysis

Total RNA was isolated from cell or tissue samples using a RNAiso Plus kit (Takara; Dalian, China; Catalog no. 9108) according to the manufacturer's instruction. A total of 1 µg RNA was reverse transcribed into cDNA using a PrimeScript RT reagent kit and treated with the gDNA Eraser (Takara; Catalog no. RR047) to remove genomic DNA according to the manufacturer's protocol. qRT‐PCR was performed on a Real‐Time PCR Instrument (Roche LightCycler 96; Switzerland) using the SYBR Premix Ex Taq (Takara; Catalog no. RR420) to detect each of the target genes. The list of qRT‐PCR primers used in this study is available in Table S3, Supporting Information. The following parameters were used for the qRT‐PCR: 95 °C for 10 min followed by 40 cycles at 95 °C for 30 s and 60 °C for 30 s. The results were expressed as a relative mRNA amount using the standard ΔΔCt method. The housekeeping gene *glyceraldehyde 3‐phosphate dehydrogenase* (*GAPDH*) was used as the endogenous control to normalize gene expression.

### Western Blot Analysis

The leg muscle tissues of mice were dissected and homogenized in RIPA buffer (Beyotime Inc.; Shanghai, China; Catalog no. C1002). The tissue homogenate was centrifuged at 16 200 × *g* for 15 min at 4 °C. The supernatants were collected and the total protein concentration was measured using a bicinchoninic acid assay kit (Epizyme Biotech; Shanghai, China; Catalog no. ZJ101). Protein samples were separated by sodium dodecyl sulfate–polyacrylamide gel and transferred from the gel to the polyvinylidene fluoride membrane (Millipore; MA, USA; Catalog no. IPVH00010). The membranes were blocked with 5% nonfat milk in tris buffered saline Tween 20 (TBST) buffer (50 mm Tris, 1.37 mm NaCl, 2.7 mm KCl, 0.05% Tween 20, pH 8.0) for 1 h at room temperature and then incubated with rabbit polyclonal anti‐Laminin primary antibody (1:100, Sigma‐Aldrich; Catalog no. L9393) or rabbit monoclonal anti‐GAPDH primary antibody (1:1000, Beyotime Inc.; Catalog no. AF1186) at 4 °C overnight. After washing three times with TBST buffer, the membrane was then incubated with secondary antibody (Alexa Fluor790 Goat Anti‐Rabbit, 1:5000 dilution, Jackson ImmunoResearch; PA, USA) for 1 h at room temperature. After washing three times with TBST buffer, protein bands were visualized using a fluorescent western blot imaging system (LI‐COR Odyssey Clx, USA). The protein intensity was quantified by the ImageJ software (National Institutes of Health; Bethesda, MD, USA).

### Alkaline Phosphatase Staining

iPSC colonies were fixed with 4% paraformaldehyde (PFA, Sangon Biotech; Shanghai, China; Catalog no. E672002‐0500) solution for 1–2 min and washed with PBS. Thereafter cells were stained using an AP Staining Kit (Shanghai Si Dan Race BioTechnology Co., Ltd.; China; Catalog no. 1102‐100) according to the manufacturer's instruction.

### Karyotype Analysis

iPSC_LIRE_ cells at passage 22 were grown on a T25 flask pre‐coated with 0.1% gelatin until reaching 70–80% confluency. Cells were collected and treated with 0.1 µg mL^−1^ colchicine (Sigma‐Aldrich; Catalog no. C3915) for 2.5 h. Then cells were digested with trypsin solution, re‐suspended in 0.075 m potassium chloride and incubated at 37 °C for 30 min before fixation with methanol (Sangon Biotech; CAS. 67‐56‐1) and acetic acid (Sangon Biotech Inc.; CAS. 64‐19‐7) at a volume ratio of 3:1 for 30 min at room temperature. After centrifugation, cells were re‐suspended, spread on slides, stained with Giemsa stain, air‐dried, mounted, and examined.

### Immunofluorescent Staining

Cells were fixed with 4% w/v PFA (Sangon Biotech; Catalog no. E672002‐0500) for 15 min, permeabilized with 0.2% Triton X‐100 (dissolved in PBS; Sigma; Catalog no. A600198‐0500) for 15 min, and then blocked with 10% v/v goat serum (Beyotime Inc.; Catalog no. C0265) in PBS for 30 min at room temperature. Subsequently, cells were incubated with primary antibodies overnight at 4 °C with goat polyclonal anti‐Sox2 (1:1000, R&D systems; MN, USA; Catalog no. AF2018), rabbit polyclonal anti‐Oct4 (1:400, Cell Signaling Technology; MA, USA; Catalog no. 2840), or mouse anti‐SEEA1 (1:100, Abcam; Cambridge, UK; Catalog no. ab16285), Mouse anti‐beta III tubulin (1:500, Biolegend; San Diego, USA; catalog no. MMS‐435P), or rabbit polyclonal anti‐neurofilament 200 (1:500, Sigma‐Aldrich; catalog no. N4142). After washing with PBST (0.2% Triton X‐100 in PBS) for 5 min three times, cells were incubated with secondary antibodies of rabbit anti‐mouse Alexa Fluor 488, rabbit anti‐goat Cy3, or goat anti‐rabbit Cy3 (1:200; Jackson ImmunoResearch) for 1 h at room temperature. After three 5‐min washings with PBST, the cell nuclei were counterstained with DAPI (5 µg mL^−1^, Beyotime Inc.; Catalog no. C1002) for 10 min and were imaged using an inverted fluorescence microscope (DMI8, Leica; Wetzlar, Germany).

### Immunocytochemistry

Fresh leg muscle tissues were washed three times with cold PBS and then fixed in 4% w/v PFA for 2 h at 4 °C. Tissues were cut and embedded in optimum cutting temperature compound (Jung, Leica; Catalog no. 020108926). 8 µm thick sections were prepared using a Cryostat Microtome (Leica; CM1950, Clinical Cryostat) and rinsed with PBS. The tissue sections were soaked in 0.2% Triton X‐100 for 15 min and blocked with 10% v/v goat serum in PBS for 1 h at room temperature. After incubating with the rabbit polyclonal anti‐Laminin (1:100, Sigma‐Aldrich; Catalog no. L9393) overnight at 4 °C, the slides were washed three times for 5 min each time in PBST, and then incubated with the goat anti‐rabbit Cy3 (1:200; Jackson ImmunoResearch) for 1 h at room temperature. After washing three times with PBST, the slides were incubated with DAPI solutions (5 µg mL^−1^, Beyotime Inc.; Catalog no. C1002) for 10 min at room temperature. The slides were further washed three times with PBST, sealed and observed by a fluorescence microscope (DMI8, Leica) equipped with an Olympus digital camera (Olympus DP71; Olympus).

### Teratoma Formation by iPSC_LIRE_ Cells and Hematoxylin and Eosin Staining

iPSC_LIRE_ cells were suspended at 1 × 10^7^cells/mL in PBS. 2 × 10^6^ cells were subcutaneously injected into 4‐week‐old NOD‐SCID mice (*n* = 5) obtained from Shanghai Jihui Experimental Animal Breeding Co., Ltd. (Shanghai, China). Palpable tumors were observed typically at 4 weeks after injection and surgically dissected for histological analysis. The tissues were embedded in paraffin wax (Sigma‐Aldrich; Chemical Abstracts Service [CAS] no.8002‐74‐2) and were cut into 8 µm thick sections with a microtome (Thermo Fisher Scientific, Microm HM3550). After the tissue sections were dehydrated by increasing alcohol concentrations, the tissue sections were cleared using xylene (Sangon Biotech; CAS: 1330‐20‐7) and stained with the hematoxylin staining solution (Sangon Biotech; Catalog no. E607317) for 8 min and washed in running tap water for 10 min. Next, the tissue sections were differentiated in 1% acid alcohol for 10 s, washed in running tap water for 5 min, and then were counterstained in the eosin staining solution (Sangon Biotech; Catalog no. E607321) for 30 s to 1 min and washed in running tap water for 5 min. Finally, the tissue sections were sealed by a drop of mounting medium over the tissue and then covered by a coverslip. The slides were then observed by a BX53 upright microscope (Olympus, Japan) equipped with an Olympus digital camera.

### Library Preparation and Illumina Hiseq Xten Sequencing

RNA‐seq transcriptome library was prepared following the TruSeq RNA sample preparation kit from Illumina (San Diego, CA, USA) using 5 µg of total RNA. Shortly, messenger RNA was isolated according to the polyA selection method by oligo (dT) beads and then fragmented by the fragmentation buffer. Subsequently, double‐stranded cDNA was synthesized using a SuperScript double‐stranded cDNA synthesis kit (Invitrogen, CA, USA) with random hexamer primers (Illumina). Then the synthesized cDNA was subjected to end‐repair, phosphorylation, and “A” base addition according to the Illumina's library construction protocol. Libraries were selected for cDNA target fragments of 200–300 bp on 2% Low Range Ultra Agarose followed by PCR amplified using Phusion DNA polymerase (New England Biolabs [Beijing] Ltd.; China) for 15 PCR cycles. After quantified by TBS380, paired‐end RNA‐seq sequencing library was sequenced with the Illumina HiSeq Xten (2 × 150 bp read length).

### Read Mapping

The raw paired end reads were trimmed and quality controlled by the SeqPrep (https://github.com/jstjohn/SeqPrep) and the Sickle (https://github.com/najoshi/sickle) with default parameters. Then clean reads were separately aligned to reference genome with orientation mode using the TopHat (http://tophat.cbcb.umd.edu/, version2.0.0) software.^[^ ^67^ ^]^ The mapping criteria of bowtie was as follows: sequencing reads should be uniquely matched to the genome allowing up to two mismatches, without insertions or deletions. Then the region of gene was expanded following depths of sites and the operon was obtained. In addition, the whole genome was split into multiple 15 kb windows that share 5 kb. New transcribed regions were defined as more than two consecutive windows without overlapped region of gene, where at least two reads mapped per window in the same orientation.

### Optogenetic Cell Differentiation

For neural induction, the iPSC_LIRE_ cells were transduced with lentivirus containing dCas9 (pXS204 [LTR‐P_EF1a_‐dCas9‐T2A‐Blast‐LTR]), pXS205 (LTR‐P_hCMV*‐1_‐MCP‐VPR‐T2A‐Hygro‐LTR), and two sgRNAs (pWS74 [LTR‐P_U6_‐sgRNA1 [*NEUROG2*]‐LTR], pWS76 [LTR‐P_U6_‐sgRNA2 [*NEUROG2*]‐LTR]) targeting *NEUROG2* (Table S4, Supporting Information) on 1st day after seeding into 24‐well gelatin‐coated cell culture plates in the presence of 1 µg mL^−1^ puromycin and 100 µg mL^−1^ zeocin. Transduced cells were incubated at 37 °C in 5% CO_2_ with pulsing light (460 nm, 1.0 mW cm^−2^, 1 min on, 5 min off, alternating) for 4 days or in the dark. Fresh growth medium without LIF was changed daily for 8 days. In the last 4 days, 5 × 10^−7^ m of all‐trans RA (Sigma‐Aldrich; Catalog no. R2625) was added into the media. The cells were then analyzed by qRT‐PCR.

### Calcium Ion Imaging

After differentiation, the neuronal cells were washed with Hanks’ balanced salt solution (1× HBSS) three times and then stained with 3 µm Fluo‐4 AM solution (Cat. no. F14201) for 60 min at 37 °C. After removing the Fluo‐4 AM solution and the cells were washed three times with 1× HBSS. The cells were exposed to a 50 mm KCl solution before imaging using a microscope with a rotating disc laser confocal 3D visualization system (DF505, Andor Technology). Time lapse imaging was performed at an excitation wavelength of 488 nm for 5 min. The data was analyzed using the Leica LAS AF software.

### Optogenetic Chimeric Mice Generation

For blastocyst injection, iPSC_LIRE_ cells were expanded without feeders. All mouse embryo microinjection and transplantation experiments were performed in Shanghai Model Organisms Center, Inc. Before injection, iPSC_LIRE_ cells were trypsinized and re‐suspended in M2 medium (Sigma‐Aldrich; Catalog no. M7167) and the cell suspension was incubated at 4 °C for at least 30 min. The mouse embryos were flushed out of the uterus of pregnant C57BL/6J mice with M2 medium and transferred to M2 droplets covered with mineral oil for a follow‐up injection. After injection, the blastocysts were cultured in a complete DMEM medium without LIF for about 1 h. The injected embryos were transferred to the uterus of a 2.5‐day pseudopregnant female mouse. Chimeric mice could be identified by the mosaic coat color.

### Optogenetic Activation of Gene Expression in Chimeric Mice

For exogenous gene activation experiments, the chimeric mice were hydrodynamically injected with plasmid pAF101 (P_hCMV*‐1_‐Luciferase‐pA, 300 µg) in 2 mL (10% of the body weight in grams) Ringer's solution (147 mm NaCl, 4 mm KCl, 1.13 mm CaCl_2_). At 8 h after plasmid injection, the mice were exposed to light pulses (460 nm, 10 mW cm^−2^, 2 min on, 2 min off, alternating) for 12 h, while the control mice were kept in the dark. For in vivo bioluminescence imaging, each mouse was intraperitoneally injected with 100 mm luciferin substrate solution (150 mg kg^−1^, Shanghai Science light Biology Science & Technology, China; catalog. no. luc001) under ether anesthesia. 5 min after luciferin injection, bioluminescence images of the mice were obtained using the IVIS Lumina II in vivo Imaging System (Perkin Elmer, USA), and the bioluminescence images were analyzed using the Living Image (version 4.3.1).

For endogenous gene activation experiments, the tibialis anterior and posterior muscles of the optogenetic chimeric mice were surgically exposed and electroporated with a total of 40 µg of plasmids for *Lama1* (pXS205, 20 µg; pGY174 [P_U6_‐sgRNA1*_Lama1_*‐pA::P_U6_‐sgRNA2*_Lama1_*‐pA::P_hCMV_‐dCas9‐pA], 20 µg) or 40 µg of plasmids for *Fst* (pXS205, 20 µg; pGY177 [P_U6_‐sgRNA1[*Fst*]‐pA::P_U6_‐sgRNA2[*Fst*]‐pA::P_hCMV_‐dCas9‐pA], 20 µg). The muscle was electroporated using the TERESA‐EPT‐I Drug Delivery Device (Shanghai Teresa Healthcare Sci‐Tech Co., Ltd., Shanghai, China) with the following parameters: 60 V (voltage), 50 ms (pulse width), 1 HZ (frequency). 18 h after electroporation, the mice were exposed to light pulses (460 nm, 10 mW cm^−2^, 2 min on, 2 min off, alternating) for 12 h each day for 2 days, while the control mice were kept in the dark. At 48 h after light illumination, the mice of each group were sacrificed and the muscular tissues were collected for further analysis.

### Ethics

All experiments involving animals were performed according to the protocol approved by the East China Normal University (ECNU) Animal Care and Use Committee and in direct accordance with the Ministry of Science and Technology of the People's Republic of China on Animal Care Guidelines. The protocol involved in this study was approved by the ECNU Animal Care and Use Committee (protocol ID: m20200333). All mice were euthanized after the completion of the experiments.

### Statistical Analysis

Unless otherwise mentioned, all in vitro data represented means ± SD of three independent biological replicates. For the animal experiments, the results were expressed as the mean ± SEM. Data were compared using an unpaired Student's *t*‐test, one‐way analysis of variance, or two‐way ANOVA followed by Tukey's tests. Differences were considered statistically significant at *p* < 0.05 (*), very significant at *p* < 0.01 (**), and extremely significant at *p* < 0.001 (***), *p* < 0.0001 (****). The Prism 6 software (version 6, GraphPad Software; La Jolla, CA, USA) was used for statistical analysis. *n* and *p* values are provided in the figure legends.

## Conflict of Interest

The authors declare no conflict of interest.

## Supporting information

Supporting Information

Supplemental Video 1

## Data Availability

The data that support the findings of this study are available in the supplementary material of this article.
