# Supplementary material for: An Optogenetic‐Controlled Cell Reprogramming System for Driving Cell Fate and Light‐Responsive Chimeric Mice
Source: Adv Sci (Weinh). 2022 Dec 11;10(4):2202858. doi: 10.1002/advs.202202858 (PMC9896073; doi:10.1002/advs.202202858)
Supplement: Supplementary file 1 — Supporting Information [file ADVS-10-2202858-s001.pdf]

ADVANCED SCIENCE
Open Access

Supporting Information

for *Adv. Sci.*, DOI 10.1002/advs.202202858

An Optogenetic-Controlled Cell Reprogramming System for Driving Cell Fate and Light-Responsive Chimeric Mice

*Meiyan Wang, Yuanxiao Liu, Ziwei Wang, Longliang Qiao, Xiaoding Ma, Lingfeng Hu, Deqiang Kong, Yuan Wang and Haifeng Ye**

Supplemental Information (SI)

An optogenetic-controlled cell reprogramming system for driving cell fate and light-responsive chimeric mice

*Meiyan Wang[1,3], Yuanxiao Liu[1,3], Ziwei Wang[1], Longliang Qiao[1], Xiaoding Ma[1], Lingfeng Hu[1], Deqiang Kong[1], Yuan Wang[2] and Haifeng Ye[1]**

[1]Shanghai Frontiers Science Center of Genome Editing and Cell Therapy, Biomedical Synthetic Biology Research Center, Shanghai Key Laboratory of Regulatory Biology, Institute of Biomedical Sciences and School of Life Sciences, East China Normal University, Dongchuan Road 500, Shanghai 200241, China

[2]Department of Animal Science, Michigan State University, East Lansing, Michigan, USA

[3]These authors contributed equally to this work.

*Corresponding author: Tel: +86 021 54341058; E-mail: hfye@bio.ecnu.edu.cn

Supplementary figures and tables:

Figure S1. LIRE-induced the exogenous EGFP expression in MEFs.

Figure S2. LIRE-induced exogenous EGFP expression in MEF stable cells (MEF_{liTetR}).

Figure S3. Comparison of gene activation between LIRE system and the CPTS and split-CPTS 2.0 system in MEF cells.

Figure S4. LIRE system-mediated transcription activation of endogenous *Sox2* and *Oct4* in MEF_{liTetR} stable cells.

Figure S5. Representative fluorescence microscopy images of pluripotency markers.

Figure S6. Light inducible reprogramming MEF_{LIRE} cells to $iPSC_{LIRE}$ cells using LIRE system.

Figure S7. LIRE-induced exogenous Gaussia Luciferase (Gluc) production in $iPSC_{LIRE}$ cells.

Figure S8. Light-induced endogenous *Sox2* and *Oct4* gene transcription in $iPSC_{LIRE}$ cells.

Figure S9. Transcriptional analysis of MEFs, different $iPSC_{LIRE}$ colonies and positive control ESCs.

Figure S10. Representative fluorescence microscopy images of pluripotency markers in the $iPSC_{LIRE}$.

Figure S11. Long-term performance of LIRE-induced exogenous Gaussia Luciferase (Gluc) production in $iPSC_{LIRE}$ cells.

Figure S12. Karyotypic analysis.

Figure S13. Teratoma formation analysis of the immunodeficient NOD-SCID mice.

Figure S14. LIRE-induced Gluc production in teratoma-derived fibroblasts.

Figure S15. *In vitro* differentiation of $iPSC_{LIRE}$ cells in response to light illumination.

Figure S16. Immunofluorescence images of the positive expression of neuronal markers neurofilament and beta III tubulin (Tuj1) of the neuronal cells derived from the $iPSC_{LIRE}$ cells.

Figure S17. The optogenetic chimera mice generated from $iPSC_{LIRE}$ cells after blastocyst transplantation.

Figure S18. qPCR analysis of light-induced activation of *Sox2* and *Oct4* transcription in leg muscles of the optogenetic chimera mice.

Figure S19. LIRE-induced the exogenous EGFP expression in the fibroblasts from the optogenetic chimera mice.

Table S1. Materials, reagents and antibodies used in this study

Table S2. Plasmids designed and used in this study

Table S3. Primers used for qPCR analysis

Table S4. Target sequences of sgRNAs

Table S5. Chimera mice generated from $iPSC_{LIRE}$ cells

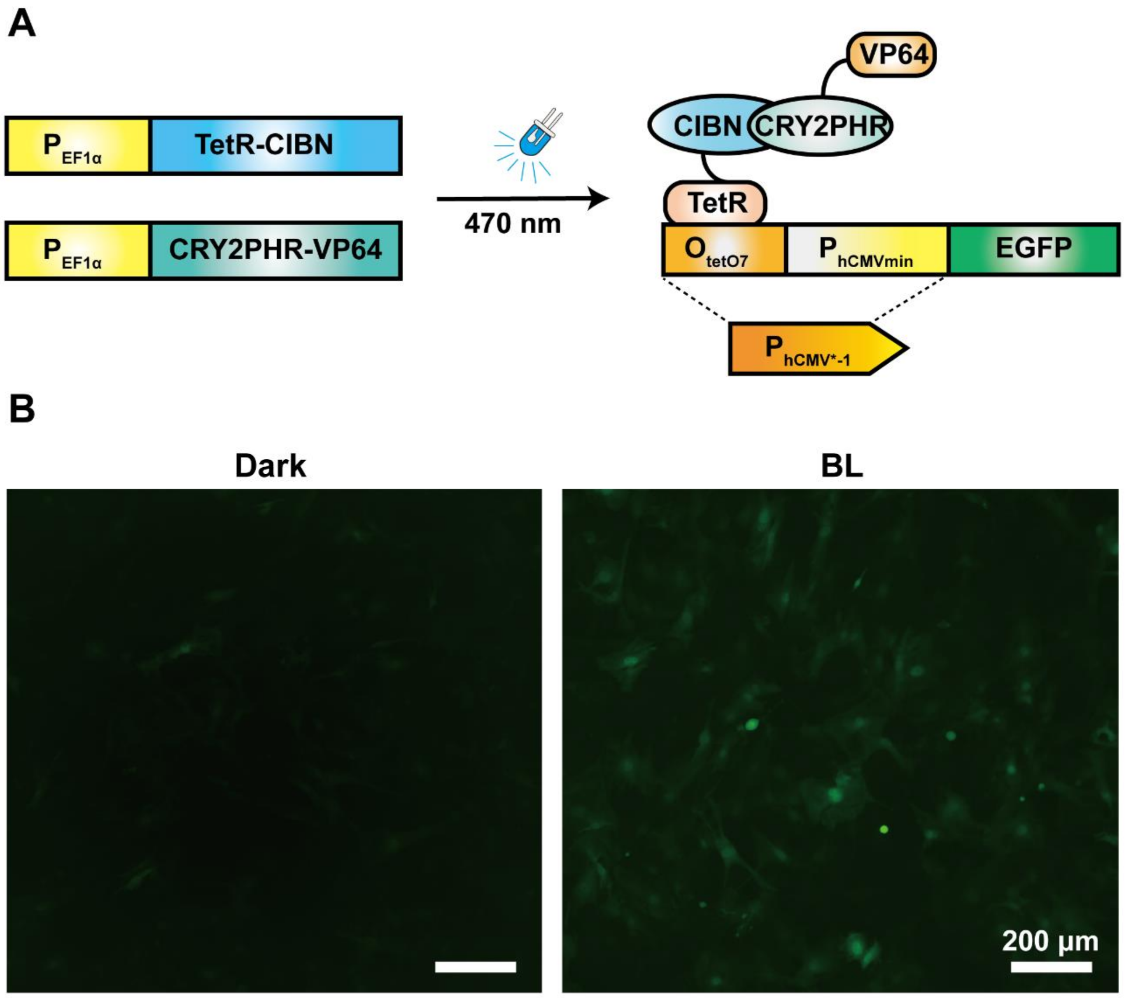

Figure S1. LIRE-induced the exogenous EGFP expression in MEFs. (**A**) Schematic representation of LIRE-induced exogenous EGFP reporter expression. N-terminal fragment of CIB1 (CIBN) from *Arabidopsis thaliana* is fused to a Tet Repressor (TetR) DNA-binding domain to create a fusion light sensor domain (CIBN-TetR) driven by a constitutive promoter (P_{EF1a}). The light-inducible heterodimerizing proteins CRY2PHR was fused to a tetrameric repeat of the minimal *Herpes simplex*-derived transactivator VP16 (VP64) to create a light-dependent transactivator (CRY2PHR-VP64) driven by a constitutive promoter (P_{EF1a}). Upon light irradiation, CRY2PHR undergoes a conformational change that enables heterodimerization with CIBN, which causes translocation of CIBN-TetR to a TetR-specific inducible promoter $P_{hCMV*-1}$ ($P_{hCMV*-1}$, tetO7-$P_{hCMVmin}$) to initiate transgene expression. (**B**) Representative fluorescence microscopy images of the light-induced exogenous EGFP expression. MEFs (2×10^4) were co-electroporated with a fusion light sensor domain

pXS173(P_{CMV}-TetR-wNES-CIBN), a light-dependent transactivator pXS172(P_{CMV}-2xNLS-CRY2PHR-NLS-VP64), and a EGFP reporter pXS170 (tetO7-$P_{hCMVmin}$-EGFP), and then illuminated with pulsing light (460 nm, 1.0 mW/cm^2) for 48 h (1 min on, 5 min off, alternating). The fluorescence of EGFP was visualized by a fluorescence microscopy 48 h after initial illumination. The images represent typical results from three independent measurements. Scale bar, 200 μm.

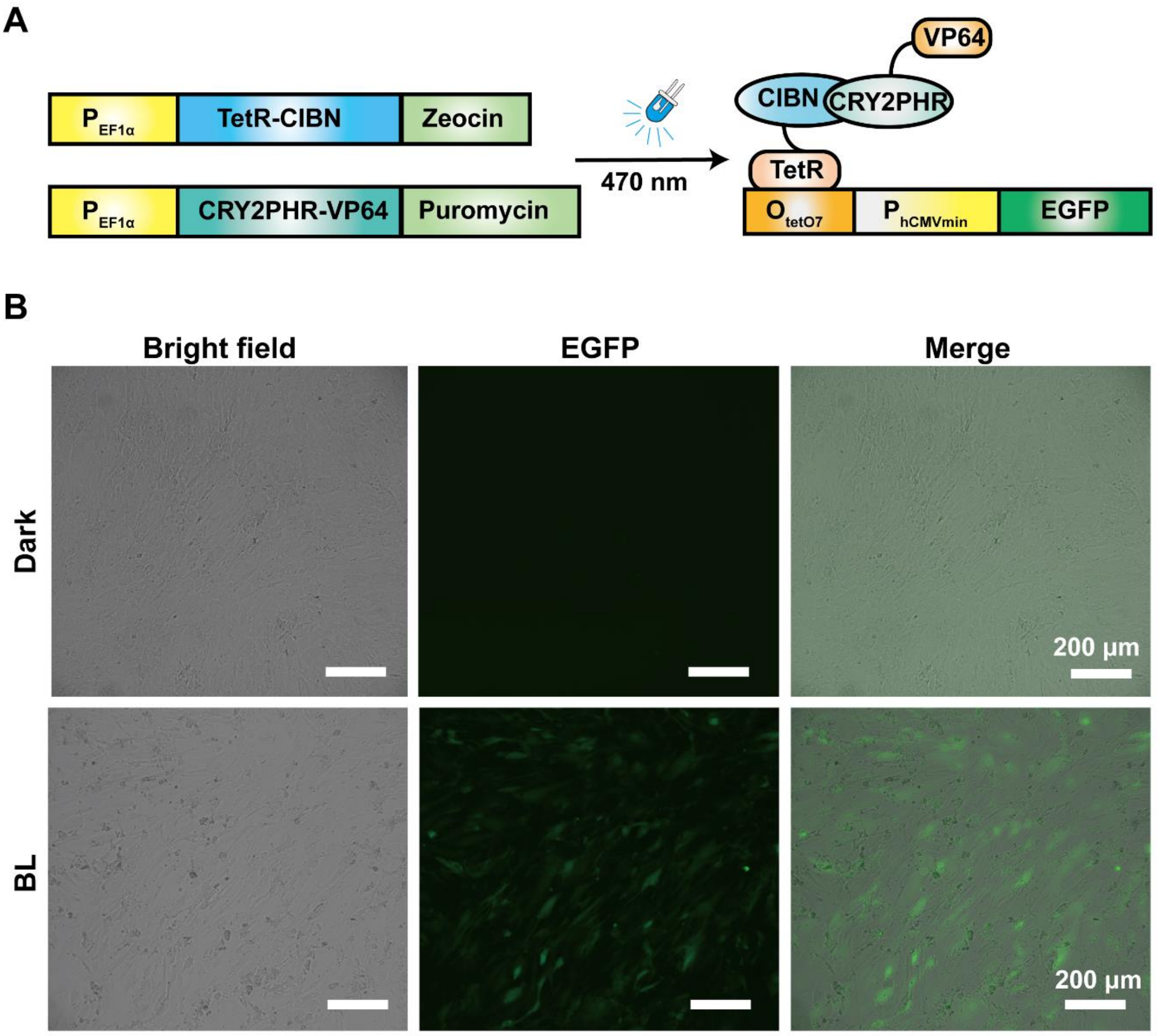

Figure S2. LIRE-induced exogenous EGFP expression in MEF stable cells (MEF$_{liTetR}$). (**A**) Schematic diagram showing light-induced exogenous EGFP reporter in MEF stable cells integrated with LIRE system (MEF$_{liTetR}$) comprising the light-dependent transactivator [lentiviral pXS206 (LTR-P$_{EF1\alpha}$-CRY2PHR-VP64-T2A-Puromycin-LTR) and the fusion light sensor domain [lentiviral pXS207 (LTR-P$_{EF1\alpha}$-TetR-CIBN-T2A-Zeocin-LTR)]. (**B**) Representative fluorescence microscopy images of the light-induced exogenous EGFP expression in MEF$_{liTetR}$ cells. MEF$_{liTetR}$ cells (2×10^4) were transduced with lentiviral pXS170 (LTR-P$_{hCMV*\text{-}1}$-EGFP-WPRE-LTR), and then illuminated with pulsing light (460 nm, 1.0 mW/cm^2) for 48 h (1 min on, 5 min off, alternating). The fluorescence of EGFP was visualized by fluorescence microscopy 48 h after light illumination. The images represent typical results from three independent measurements. Scale bar, 200 μm.

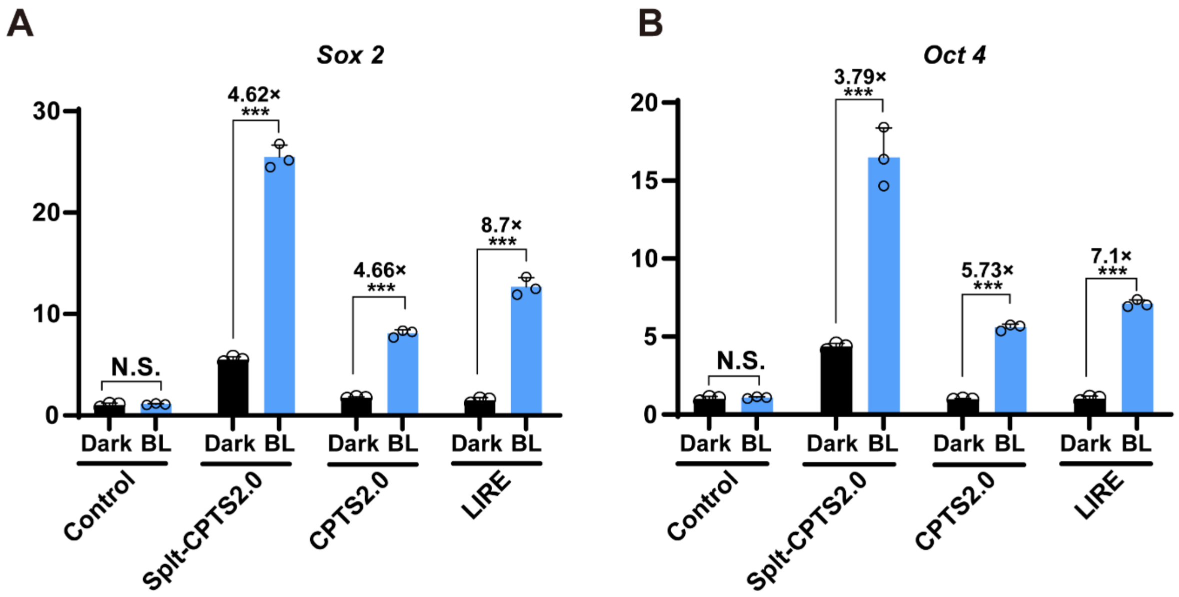

Figure S3. Comparison of gene activation between LIRE system and the CPTS 2.0 and the split-CPTS 2.0 system in MEF cells. Activation of the endogenous *Sox2* (**A**) and *Oct4* (**B**) using LIRE,split-CPTS 2.0 and CPTS 2.0. MEFs (1×10^5) were co-electroporated with the LIRE system [pXS206, pXS207, pXS204 (LTR-$P_{EF1\alpha}$-dCas9-T2A-Blasticidin-LTR), pXS205 (LTR-$P_{hCMV*-1}$-MCP-VPR-T2A-Hygro-LTR)] or CPTS 2.0 (pCMV-NLSdCas9-NLS, pCMV-NLSx3-MS2-CIB1, pCMV-NLSx3-CRY2PHR-p65-HSF1) or Split-CPTS2.0 (pCMV-NES-dCas9N-pMag-NES, pCMV-nMagHigh1-dCas9C-NLS-VP64, pCMV-NLS-MS2ΔFG-NLS-p65-HSF1), and gRNA targeting the *Sox2* and *Oct4* locus (pS3-O2*)* at a 1:1:1:1:1 ratio, and then illuminated with pulsing light (460 nm, 1.0 mW/cm^2) for 48 h (1 min on, 5 min off, alternating), and the relative mRNA expression of *Sox2* and *Oct4* was quantified by qPCR. The data are expressed as the amount of mRNA relative to the negative control cells in the dark. Data represent the mean ± SD ($n = 3$ independent experiments) and were analyzed by Student's *t*-test. ***$P < 0.001$, N.S., not significantly different.

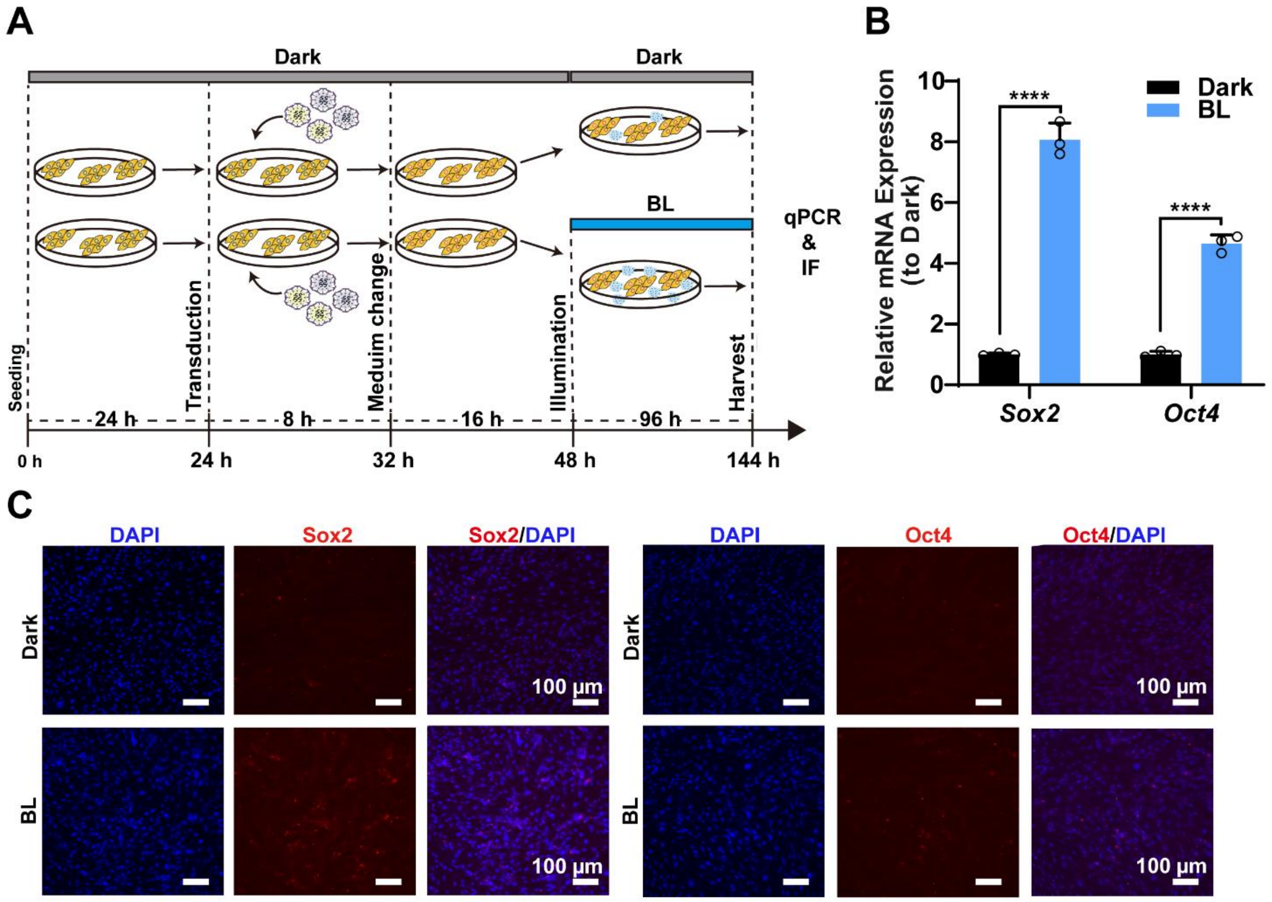

Figure S4. LIRE system-mediated transcription activation of endogenous *Sox2* and *Oct4* in MEF$_{LIRE}$ stable cells. (**A**) Schematic timeline of seeding, transfection, medium change and light illumination for LIRE system-mediated transcription activation in MEF$_{LIRE}$ stable cells. (**B**) The endogenous *Sox2* and *Oct4* activation by LIRE system. MEF$_{LIRE}$ stable cells (2×10^4) were co-transduced with lentiviral pXS204 (LTR-P$_{EF1\alpha}$-dCas9-T2A-Blasticidin-LTR), pXS205 (LTR-P$_{hCMV*-1}$-MCP-VPR-T2A-Hygro-LTR), and pS3-O2 (LTR-P$_{U6}$-S84-P$_{U6}$-S136-P$_{U6}$-S148-P$_{U6}$-O71-P$_{U6}$-O127-LTR), and then illuminated with pulsing light (460 nm, 1.0 mW/cm^2) for 4 d (1 min on, 5 min off, alternating), and the relative mRNA expression of *Sox2* and *Oct4* was quantified by qPCR. The data are expressed as the amount of mRNA relative to the dark controls. (**C**) Representative fluorescence microscopy images of the light inducible endogenous Sox2 and Oct4 transcription activation. Red indicates endogenous Sox2 and Oct4 expression; Blue indicates DAPI staining nuclei. The images represent typical results from three independent measurements. Scale bar, 100 μm. Data in B represent the mean ± SD ($n = 3$ independent experiments) and were analyzed by Student's *t*-test. ****$P < 0.0001$.

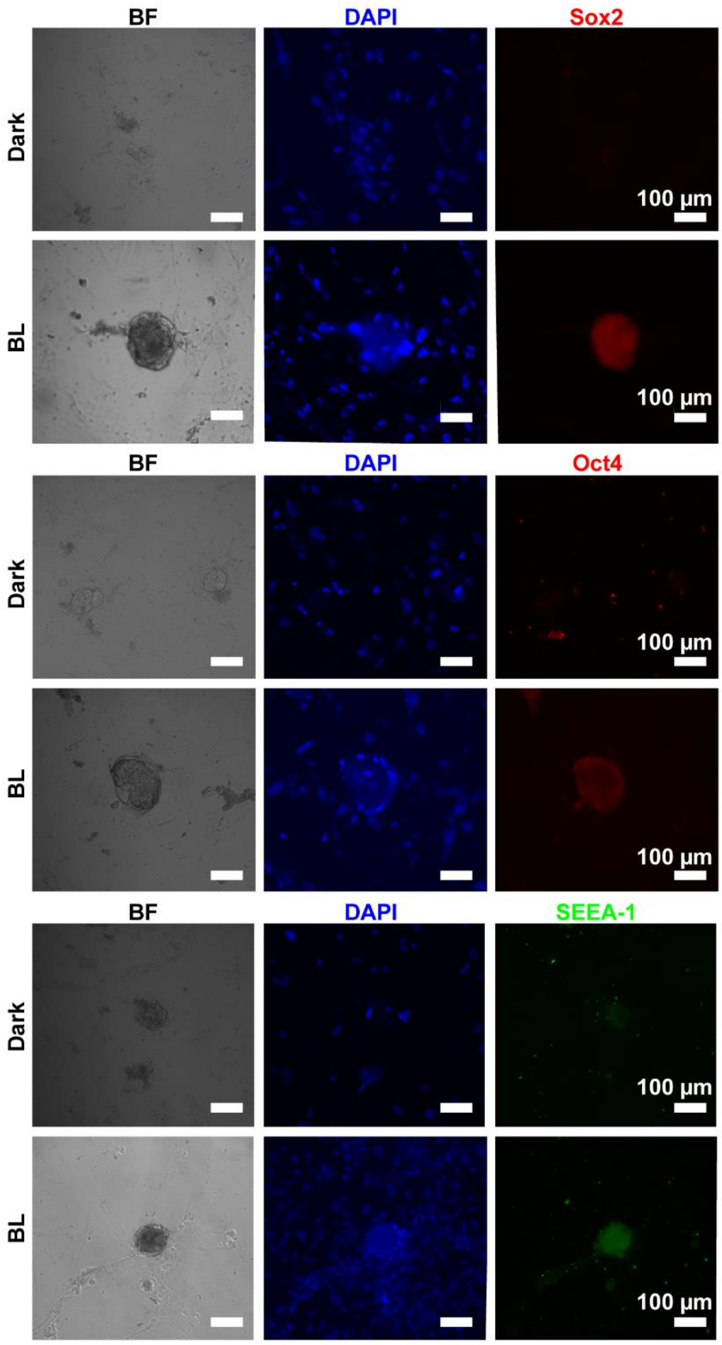

Figure S5. Representative fluorescence microscopy images of pluripotency markers. The pluripotency markers: Sox2 (Red), Oct4 (Red), and SSEA-1 (Green) were observed in iPSC$_{LIRE}$ colonies derived from light-inducible MEFs at day 21. Blue indicates DAPI staining of nuclei, BF indicates bright field image. Scale bar, 100 µm.

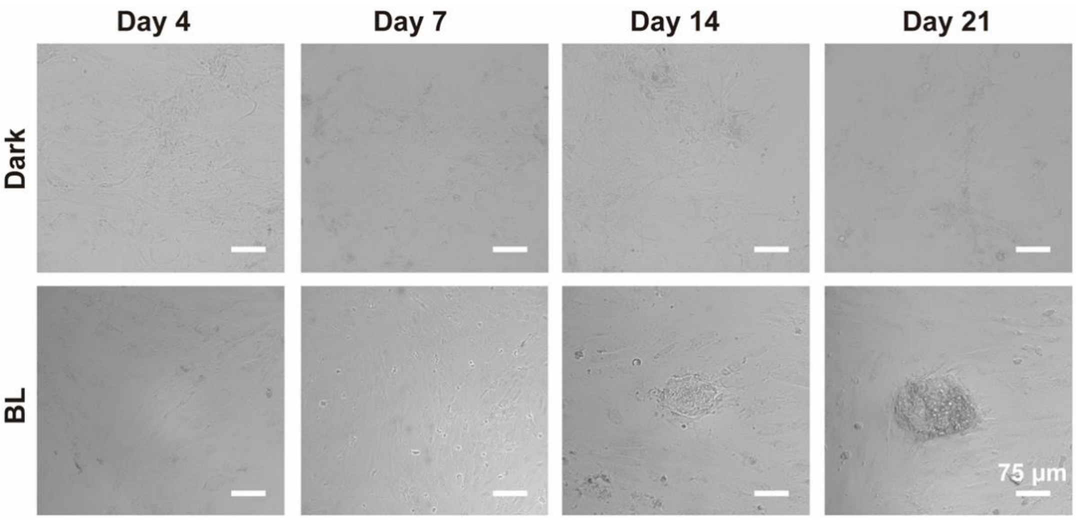

Figure S6. Light inducible reprogramming MEF$_{LIRE}$ cells to iPSC$_{LIRE}$ cells using LIRE system. MEF$_{LIRE}$ cells (2×10^4) were transduced with lentiviral pXS204 (LTR-P$_{EF1\alpha}$-dCas9-T2A-Blasticidin-LTR), pXS205 (LTR-P$_{CMV}$-MS2-VPR-T2A-Hygro-LTR), and pS3-O2 (LTR-P$_{U6}$-S84-P$_{U6}$-S136-P$_{U6}$-S148-P$_{U6}$-O71-P$_{U6}$-O127-LTR), and then illuminated with pulsing light (460 nm, 1.0 mW/cm^2) for 10 d (1 min on, 5 min off, alternating). The cell morphology was observed using bright field microscopy at day 4, 7, 14, 21 in the illuminated group cells and cells in the dark. Scale bar, 75 μm.

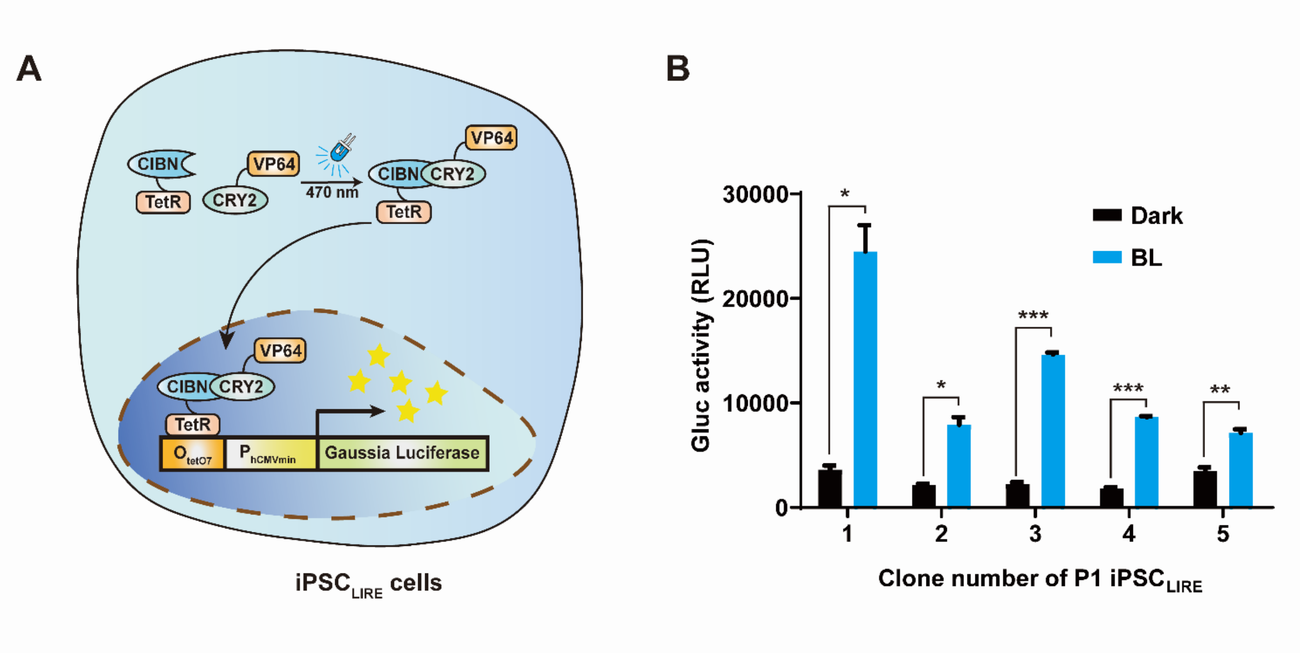

Figure S7. LIRE-induced exogenous Gaussia Luciferase (Gluc) production in iPSC$_{LIRE}$ cells. (**A**) Schematic representation of iPSC$_{LIRE}$ cells stably integrated with the LIRE system. Upon light irradiation, CRY2PHR undergoes a conformational change that enables heterodimerization with CIBN, which causes translocation of CIBN-TerR to a TetR-specific inducible promoter $P_{hCMV*-1}$ ($P_{hCMV*-1}$, tetO7-$P_{hCMVmin}$) to activate Gluc reporter expression. (**B**) Light-inducible secretion of Gluc in the selected iPSC$_{LIRE}$ cell clones at passages 1. The selected iPSC$_{LIRE}$ cell clones (2×10^4) were transduced with lentiviral pLX78 (LTR-$P_{hCMV*-1}$-Gluc-WPRE-LTR), and then illuminated with pulsing light (460 nm, 1.0 mW/cm^2) for 48 h (1 min on, 5 min off, alternating). Gluc activity in the culture supernatant was measured 48 h after light illumination. Data in B represent the mean ± SD (n = 3 independent experiments) and were analyzed by Student's t-test. *$P < 0.05$, **$P < 0.01$, ***$P < 0.001$.

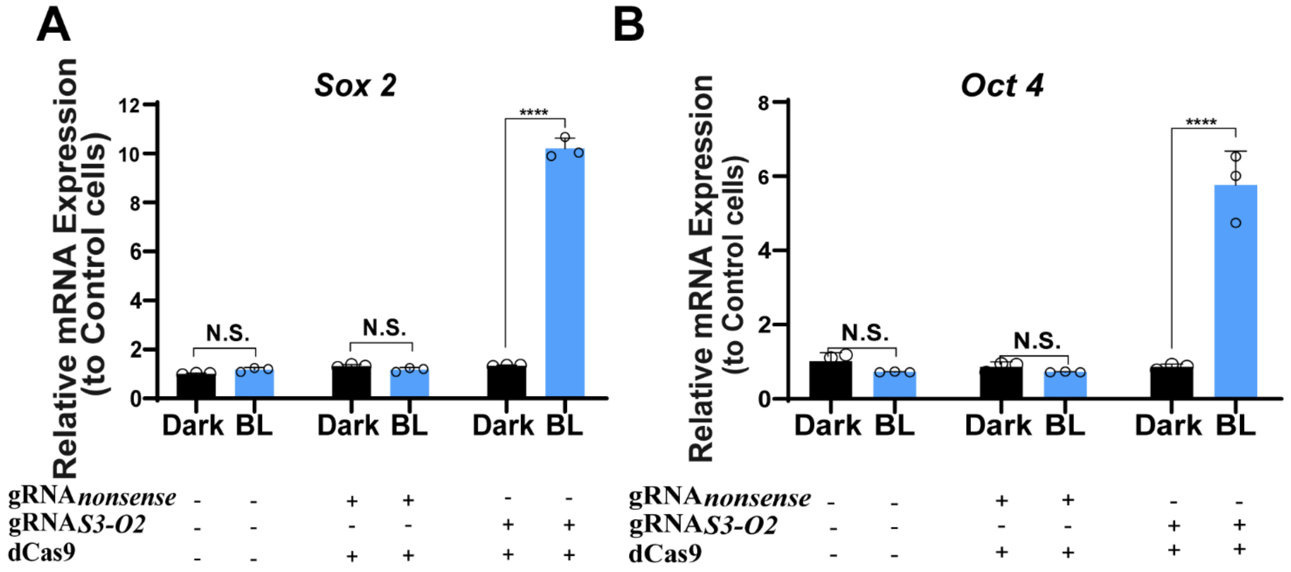

Figure S8. Light-induced endogenous *Sox2* and *Oct4* gene transcription in iPSC$_{\text{LIRE}}$ cells. iPSC$_{\text{LIRE}}$ cells (2×10^4) were transduced with lentiviral pXS204, pXS205, and gRNA targeting the *Sox2* and *Oct4* locus (pS3-O2) or a nonsense control locus (pWS68, P$_{\text{U6}}$-sgRNA$_{nonsense}$)], and then illuminated with pulsing light (460 nm, 1.0 mW/cm^2) for 48 h (1 min on, 5 min off, alternating). The relative mRNA expression of *Sox2* (A)and *Oct4* (B) was quantified by qPCR. The data are expressed as the amount of mRNA relative to the negative control cells in the dark. Data represent the mean ± SD (n = 3 independent experiments) and were analyzed by Student's t-test. ****$P < 0.0001$, N.S., not significantly different.

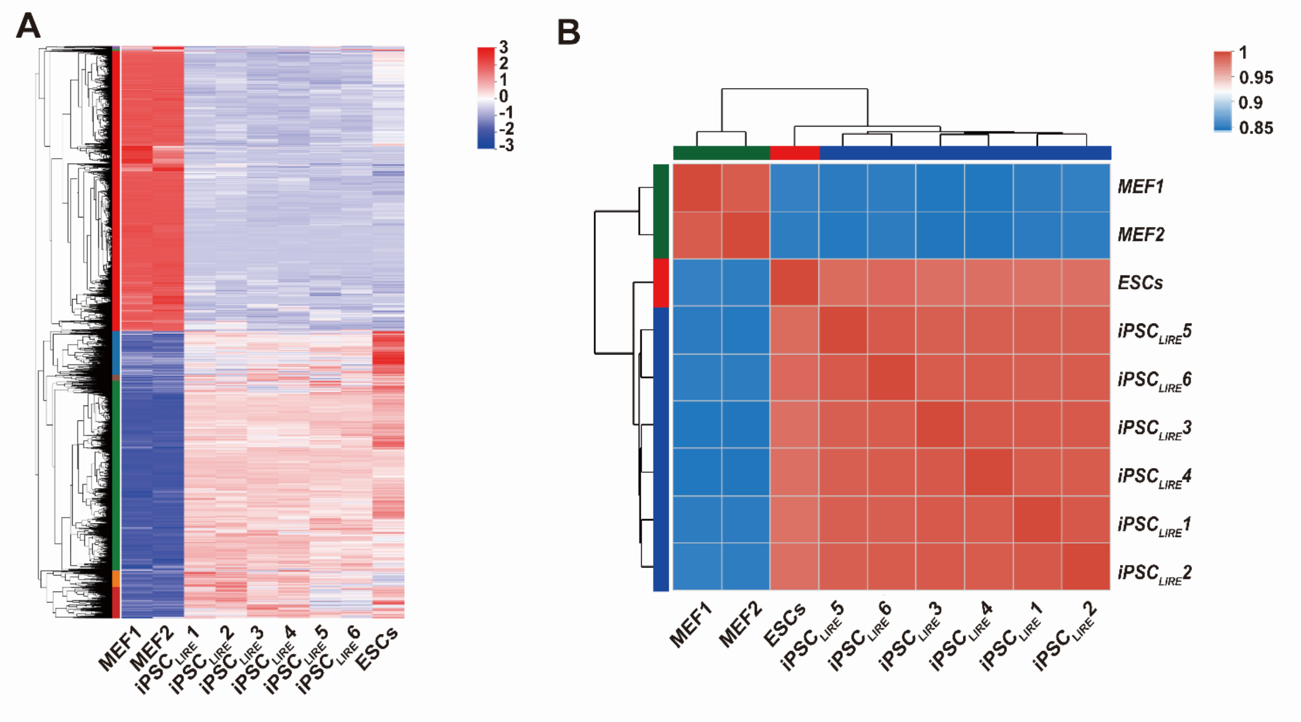

Figure S9. Transcriptional analysis of MEFs, different iPSC$_{LIRE}$ colonies and positive control ESCs. (**A**) Heatmaps representing the global expression patterns of MEFs, different iPSC$_{LIRE}$ colonies and positive control ESCs. (**B**) Correlation analysis of MEFs, different iPSC$_{LIRE}$ colonies and positive control ESCs.

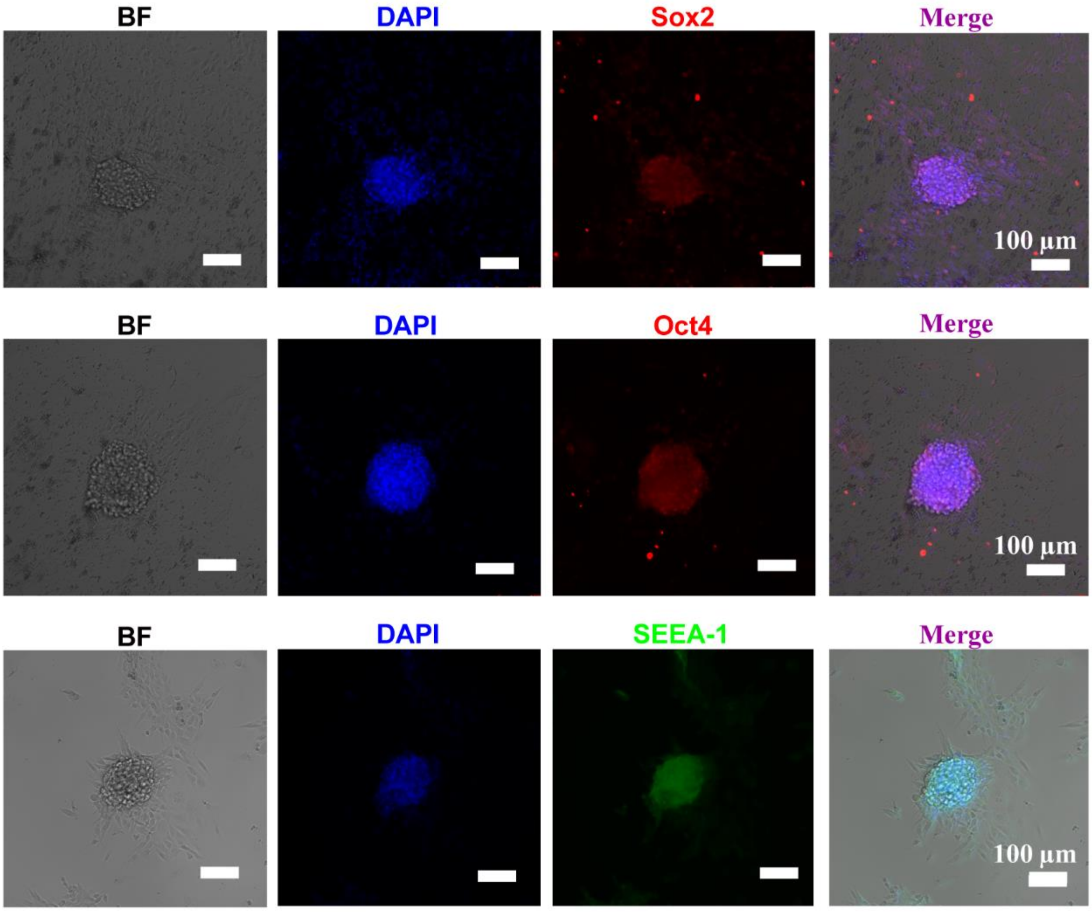

Figure S10. Representative fluorescence microscopy images of pluripotency markers in the iPSC$_{\text{LIRE}}$. Blue indicates DAPI staining of nuclei; Red indicates Sox2 and Oct4; Green indicates SSEA-1; BF indicates bright field image. These images represent typical results from two independent measurements. Scale bar, 100 µm.

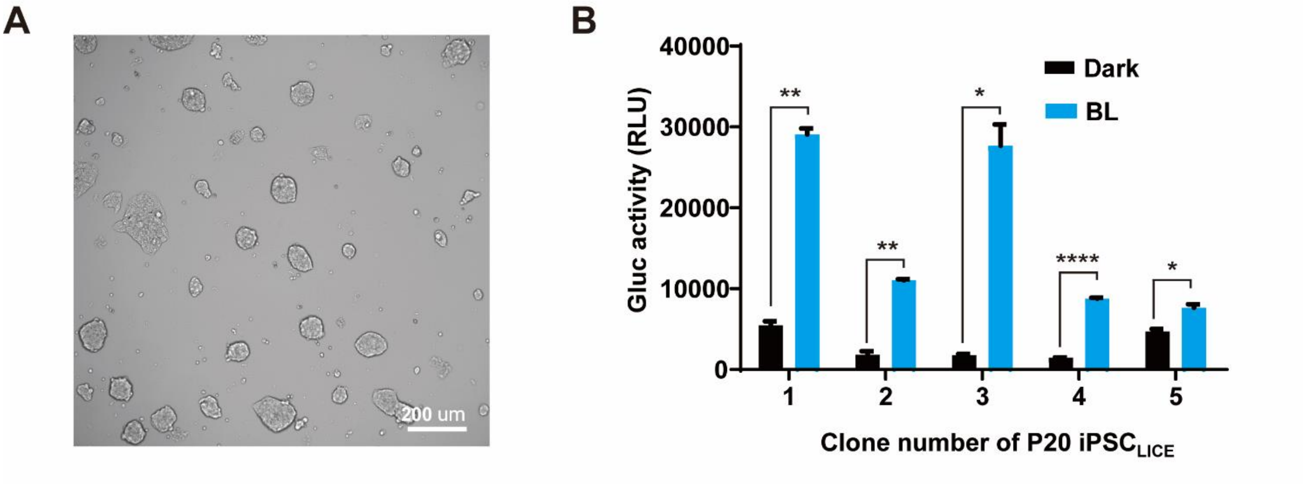

Figure S11. Long-term performance of LIRE-induced exogenous Gaussia Luciferase (Gluc) production in iPSC$_{LIRE}$ cells. (**A**) The iPSC$_{LIRE}$ cell morphology was observed using bright field microscopy at passage 20. Scale bar, 200 µm. (**B**) Light-inducible production of Gluc in the selected iPSC$_{LIRE}$ cell clones at passage 20. The selected iPSC$_{LIRE}$ cell clones (2×10^4) were transduced with lentiviral pLX78 (LTR-P$_{hCMV*-1}$-Gluc-WPRE-LTR), and then illuminated with pulsing light (460 nm, 1.0 mW/cm^2) for 48 h (1 min on, 5 min off, alternating). Gluc activity in the culture supernatant was measured 48 h after light illumination. Data in B represent the mean ± SD (n = 3 independent experiments) and were analyzed by Student's t-test. *$P < 0.05$, **$P < 0.01$, ****$P < 0.0001$.

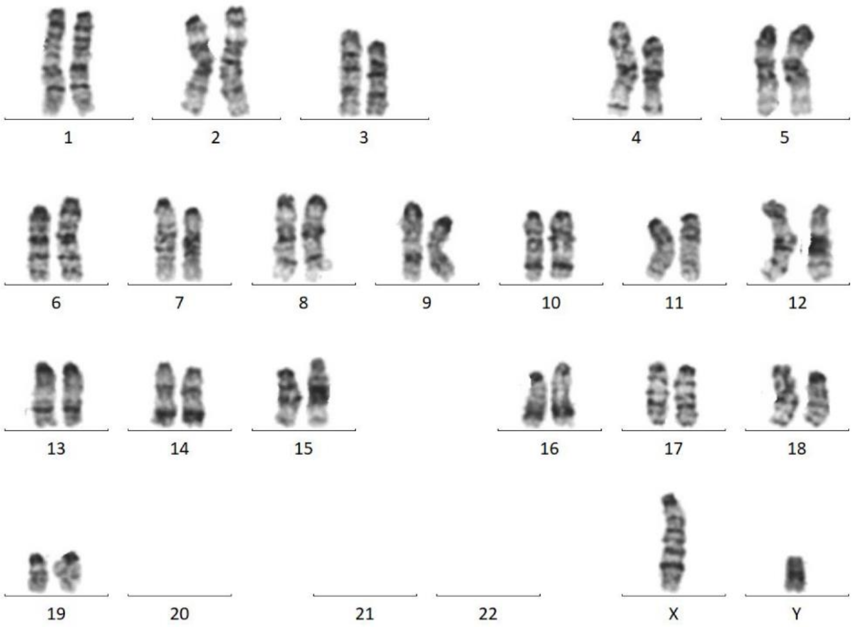

Figure S12. Karyotypic analysis. iPSC$_{LIRE}$ cells at passage 22 were grown on a T25 flask pre-coated with 0.1% gelatin until reaching 70–80% confluency. Then the cells were used for karyotype analysis. These cells showed a normal 40, XY karyotype.

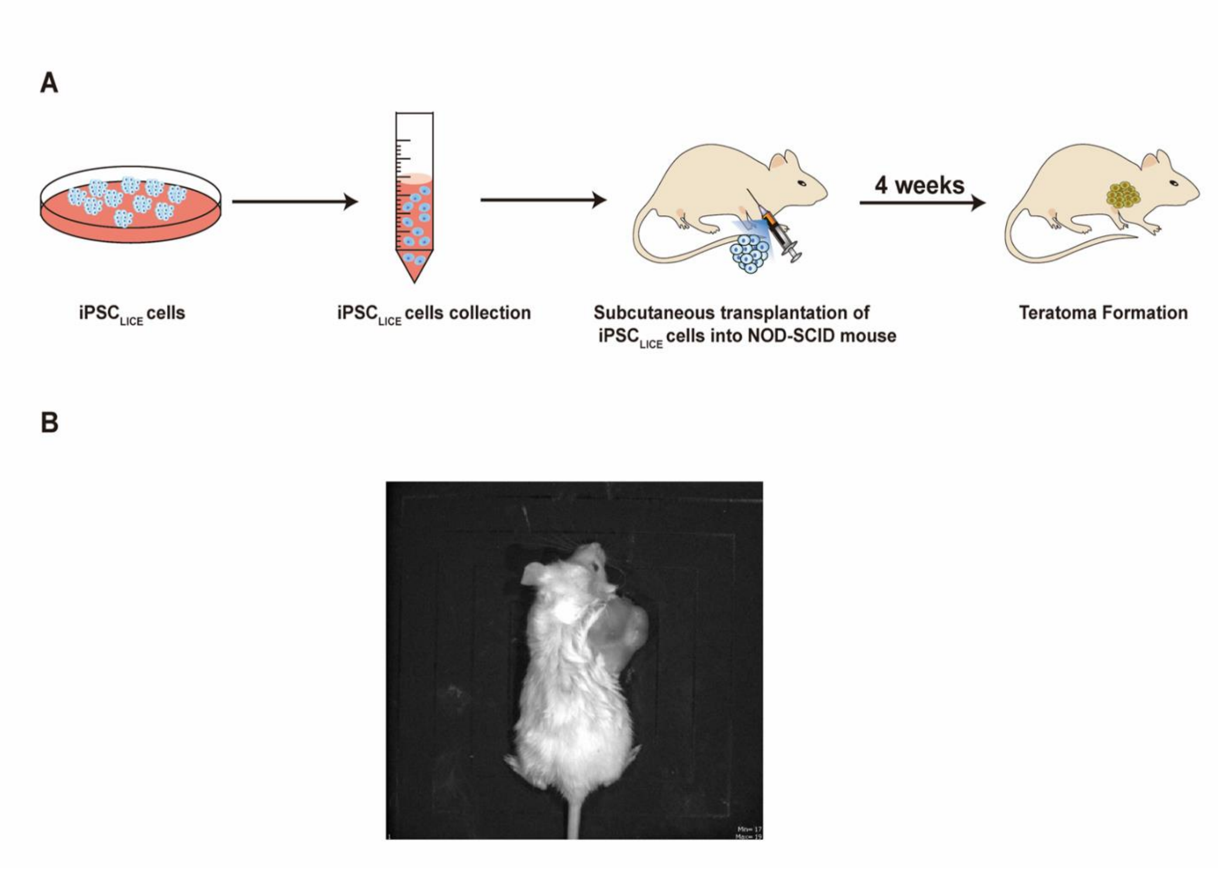

Figure S13. Teratoma formation analysis of the immunodeficient NOD-SCID mice. (**A**) Schematic representation of the experimental procedure for evaluating capability of teratoma formation of iPSCs$_{LIRE}$ cells *in vivo*. After collecting iPSCs$_{LIRE}$ cells, 3×10^6 cells were subcutaneously injected into each NOD-SCID mouse. (**B**) Photograph of NOD-SCID mice at four weeks after iPSCs$_{LIRE}$ cells injection.

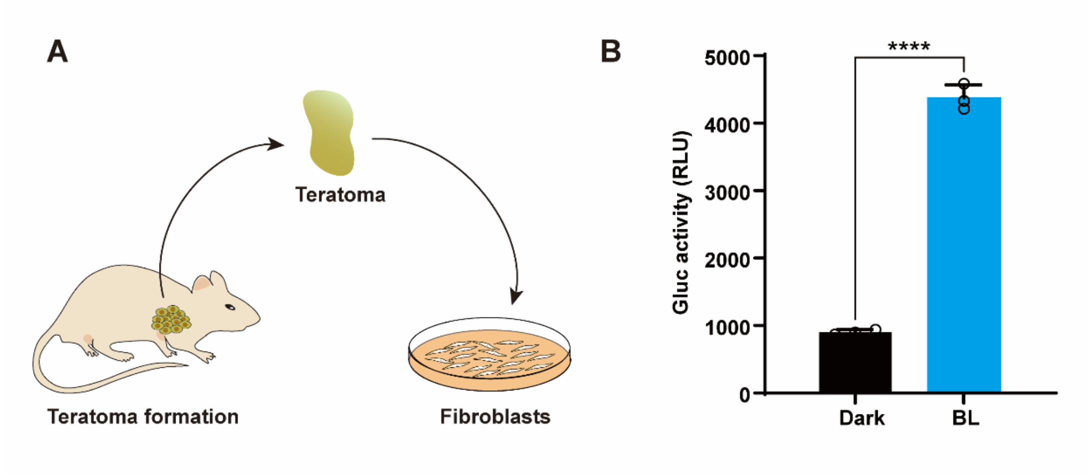

Figure S14. LIRE-induced Gluc production in teratoma-derived fibroblasts. (**A**) Schematic representation of the experimental procedure for isolating fibroblasts from iPSCs$_{LIRE}$ cells formed teratomas in NOD-SCID mouse. (**B**) Light-inducible production of Gluc in the culture supernatant of fibroblasts isolated from iPSCs$_{LIRE}$ cells formed teratomas. The fibroblasts cells (2×10^4) were transduced with lentiviral pLX78 (LTR-P$_{hCMV*-1}$-Gluc-WPRE-LTR]), and then illuminated with pulsing light (460 nm, 1.0 mW/cm^2) for 48 h (1 min on, 5 min off, alternating). Gluc activity in the culture supernatant was measured 48 h after light illumination. Data in B represent the mean ± SD (*n* = 3 independent experiments) and were analyzed by Student's *t*-test. ****$P < 0.0001$.

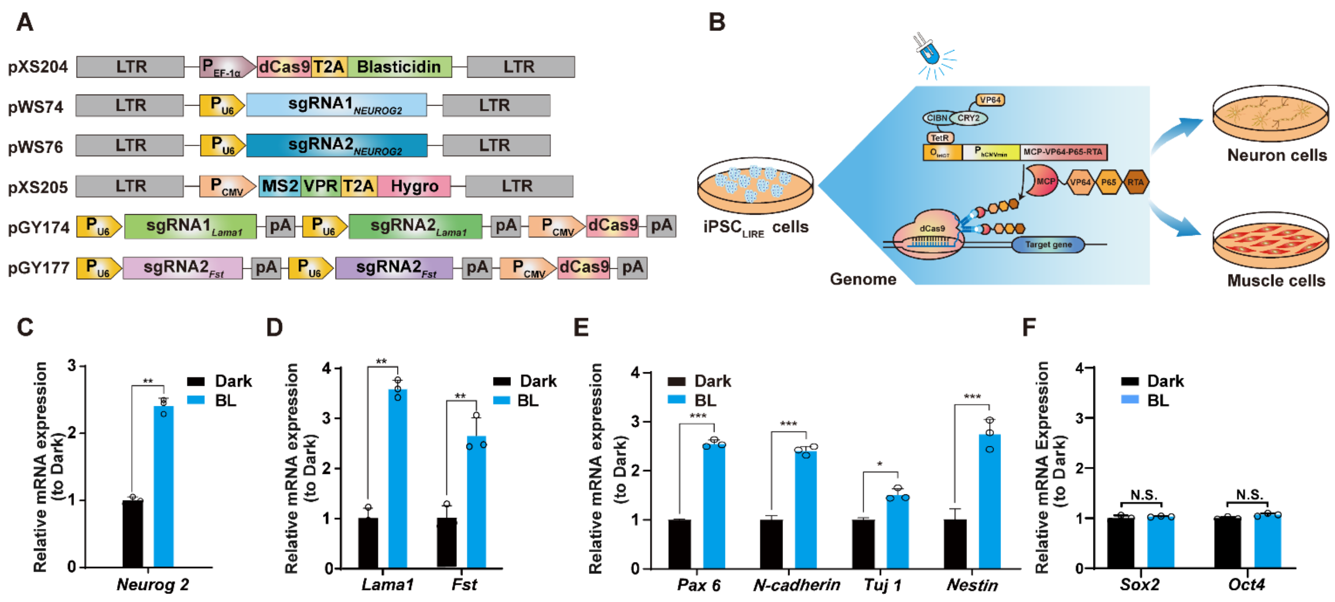

Figure S15. ***In vitro*** **differentiation of iPSC$_{LIRE}$ cells in response to light illumination.** (**A**) Schematic depicting the genetic configuration of constructs used for iPSC$_{LIRE}$ cells differentiation. (**B**) Schematic representation of light-induced neuronal or muscle differentiation from iPSC$_{LIRE}$ cells. (**C**) Light-induced endogenous *NEUROG2* upregulation in iPSC$_{LIRE}$ cells. iPSC$_{LIRE}$ cells (2×10^4) were transduced with lentiviral pXS204 (LTR-PEF1α-dCas9-T2A-Blasticidin-LTR), pXS205 (LTR-P$_{CMV}$-MS2-VPR-T2A-Hygro-LTR), and two sgRNAs targeting *NEUROG2* [pWS74(LTR-P$_{U6}$-sgRNA1 (*NEUROG2*)-LTR) and pWS76(LTR-P$_{U6}$-sgRNA2 (*NEUROG2*)-LTR)], and then illuminated with pulsing light (460 nm, 1.0 mW/cm^2) for 4 d (1 min on, 5 min off, alternating). The relative mRNA expression of *NEUROG2* was quantified by qRT-PCR. The data are expressed as the amount of mRNA relative to the dark controls. (**D**) light-induced endogenous *Lama1* and *Fst* upregulation in iPSC$_{LIRE}$ cells. iPSC$_{LIRE}$ cells (2×10^4) were electroporated with pXS205 (LTR-P$_{CMV}$-MS2-VPR-T2A-Hygro-LTR, 200 ng), pGY174[P$_{U6}$-sgRNA1(*Lama1*)-pA-P$_{U6}$-sgRNA2(*Lama1*)-pA-P$_{hCMV}$-dCas9-pA, 100 ng] or pGY177[P$_{U6}$-sgRNA1 (*Fst*)-pA-P$_{U6}$-sgRNA2(*Fst*)-pA-P$_{hCMV}$-dCas9-pA, 100 ng], and then illuminated as described in ***C***. The relative mRNA expression of *Lama1 and Fst* was quantified by qRT-PCR. (**E**) qRT-PCR analysis of mRNA expression levels of neuronal typical marker genes (*Pax6, N-cadherin, Tuj1* and *Nestin*) in iPSC$_{LIRE}$ cells after 8 d of light illumination. (**F**) qRT-PCR analysis of mRNA expression levels of *Sox2* and *Oct4* genes in iPSC$_{LIRE}$ cells after 8 d of light illumination. The data are expressed as the amount of mRNA relative to the dark controls. **C-F** data represent the mean ± SD ($n = 3$ independent experiments) and were analyzed by Student's t-test. $^*P < 0.05$, $^{**}P <$

0.01, ***$P < 0.001$, N.S., not significantly different.

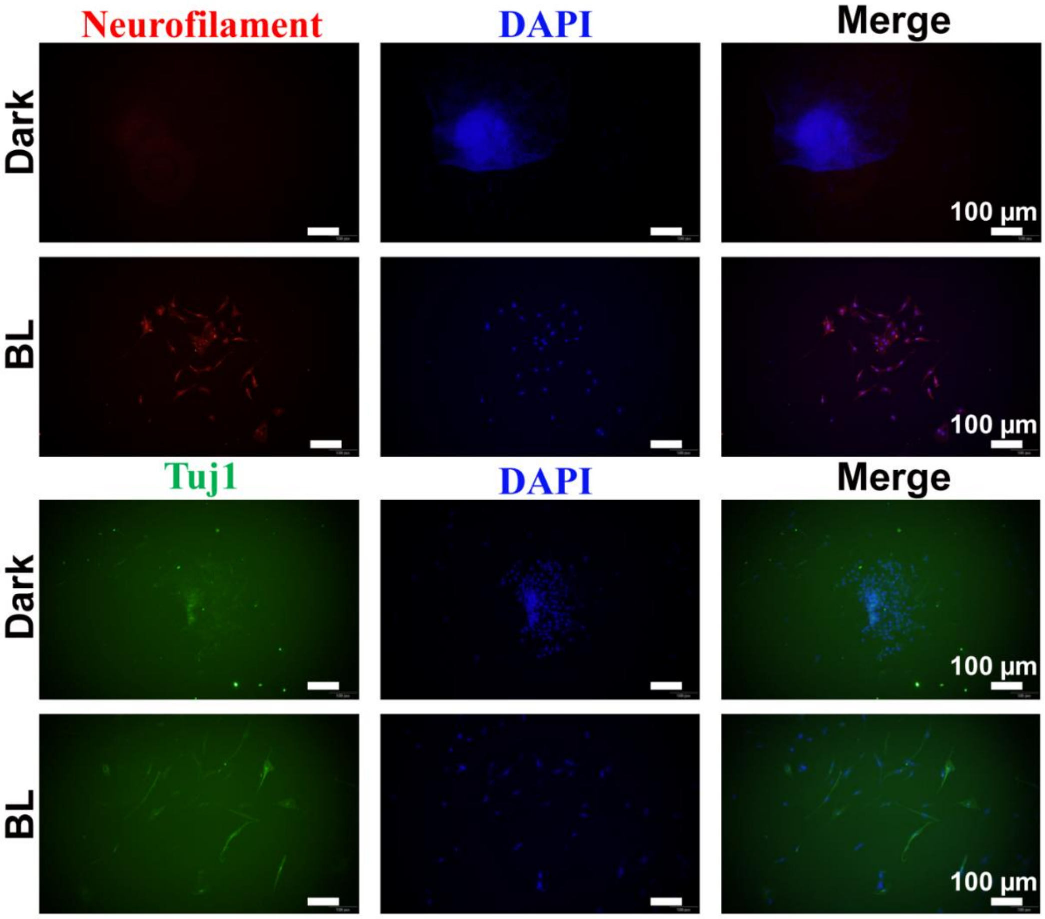

Figure S16. Immunofluorescence images of the neuronal markers neurofilament and beta III tubulin (Tuj1) of the neuronal cells derived from the iPSC$_{LIRE}$ cells. Blue indicates DAPI staining of nuclei; Red indicates neurofilament; Green indicates beta III tubulin (Tuj1). The images represent typical results from two independent measurements. Scale bar, 100 µm.

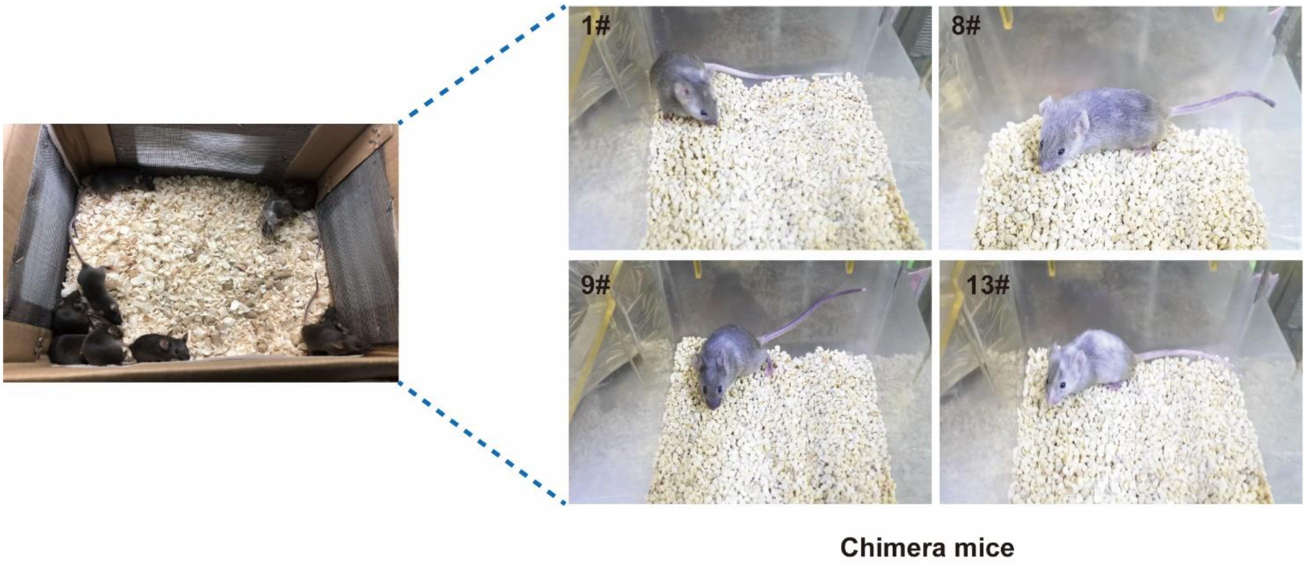

Figure S17. The optogenetic chimera mice generated from iPSC$_{LIRE}$ cells after blastocyst transplantation. Photograph of the optogenetic chimera mice generated from iPSC$_{LIRE}$ cells. 1#, 8#, 9#, 13#: Chimeric mice ID.

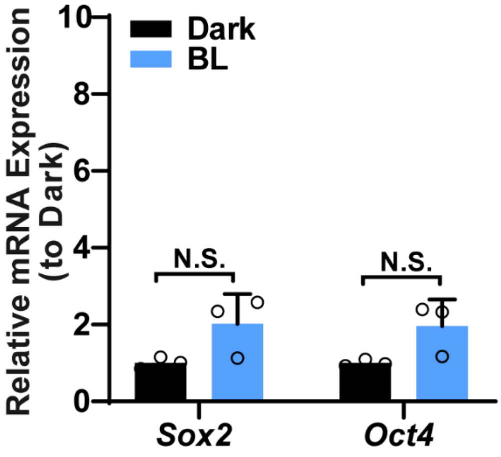

Figure S18. qPCR analysis of light-induced activation of *Sox2* and *Oct4* transcription in leg muscles of the optogenetic chimera mice. The muscles of the mice were electroporated with 40 μg of BL-inducible transactivator vector pXS205 (LTR-$P_{hCMV*-1}$-MCP-VPR-T2A-Hygro-LTR) and the concatenated dCas9 and sgRNA1 vector pGY174[P_{U6}-sgRNA1(*Lama1*)-pA-P_{U6}-sgRNA2(*Lama1*)-pA-P_{hCMV}-dCas9-pA] or pGY177[P_{U6}-sgRNA1 (*Fst*)-pA-P_{U6}-sgRNA2(*Fst*)-pA-P_{hCMV}-dCas9-pA], and the mice were either exposed to blue light (460 nm, 10 mW/cm^2) for 8 h/day for two days (2 min on, 2 min off, alternating) or kept in the dark. Three days after electroporation, muscle tissues were harvested and the relative mRNA expression of *Sox2* and *Oct4* was quantified by qRT-PCR. Data represent the mean ± SEM (n = 4 mice). P values were calculated by Student's t-test. N.S., not significantly different.

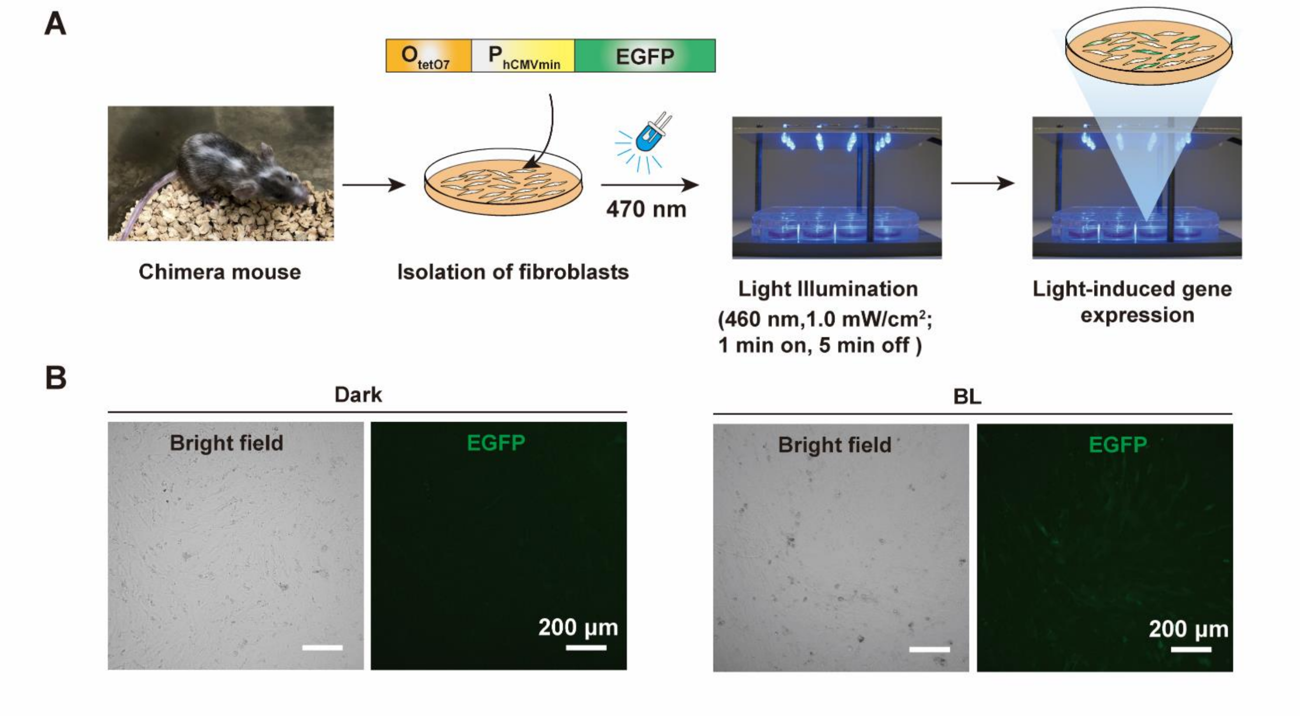

Figure S19. LIRE-induced the exogenous EGFP expression in the fibroblasts from the optogenetic chimera mice. (**A**) Schematic representation of the experimental procedure for isolating fibroblasts from chimeric mice and light-inducible gene expression, only by utilizing a beam of light. (**B**) Representative fluorescence microscopy images of the light-induced the exogenous EGFP expression in the fibroblasts from the chimeric mice. The fibroblasts cells (2×10^4) were transduced with lentiviral reporter pXS170 (LTR- $P_{hCMV*-1}$-EGFP-WPRE-LTR), and then illuminated with pulsing blue light (460 nm, 1.0 mW/cm^2) for 48 h (1 min on, 5 min off, alternating). The fluorescence of EGFP was visualized by fluorescence microscopy 48 h after light illumination. The images represent typical results from two independent measurements. Scale bar, 200 µm.

Table S1. Materials, reagents and antibodies used in this study

REAGENT OR RESOURCE	SOURCE	IDENTIFIER
**Antibodies**		
Rabbit polyclonal anti-Laminin	Sigma-Aldrich	Cat#L9393
Goat polyclonal anti-Sox2	R&D systems	Cat#AF2018
Rabbit polyclonal anti-Oct4	Cell Signaling Technology	Cat#2840
Mouse anti-SEEA1	Abcam	Cat#ab16285
**Chemicals, Peptides, and Recombinant Proteins**		
CHIR99021	Selleck	Cat#S2924
PD0325901	Selleck	Cat#S1036
Retinoic acid	Sigma	Cat#CR2625
DAPI	ThermoFisher Scientific	Cat#D1306
D-Luciferin	Sigma	Cat#L9504
RNAiso Plus kit	Takara	Cat#9108
PrimeScript RT reagent Kit	Takara	Cat#RR047
SYBR Premix Ex Taq	Takara	Cat#RR420
P3 Primary Cell 4D-Nucleofector X Kit S	Lonza	Cat#V4XP-3032
P4 Primary Cell 4D-NucleofectorTM X Kit S	Lonza	Cat#V4XP-4032
FBS	ThermoFisher Scientific	16000-044
DMEM	ThermoFisher Scientific	Cat#11995
Glasgow's MEM (GMEM)	ThermoFisher Scientific	Cat#11710035
GlutaMAX^{TM}	ThermoFisher Scientific	Cat#35050061
MEM Non-essential amino acids (NEAA)	ThermoFisher Scientific	Cat#11140050
LIF	Millipore	Cat#ESG1107
Puromycin	ThermoFisher Scientific	Cat#A1113803
Zeocin	ThermoFisher Scientific	Cat# R25001

Table S2. Plasmids designed and used in this study

**Plasmid**	**Description and Cloning Strategy**	**Reference**
pcDNA3.1(+)	Constitutive mammalian P_{hCMV}-driven expression vector (P_{hCMV}-MCS-pA)	Invitrogen' CA
pLL3.7	3rd generation lentiviral vector that expresses shRNA under the mouse U6 promoter. A P_{hCMV} -EGFP reporter cassette is included in the vector (LTR-P_{hCMV}-EGFP-WPRE-LTR).	Addgene (#11795)
sgRNA (MS2) cloning backbone	sgRNA cloning backbone with MS2 loops at tetraloop and stem loop 2, containing *Bbs*I site for insertion of spacer sequences	Addgene (#61424)
MS2-P65-HSF1_GFP	Expresses the MS2-P65-HSF1 activator helper complex with a 2A GFP	Addgene (#61423)
Lenti dCAS-VP64_Blast	3rd generation lenti vector encoding dCAS9-VP64 with 2A Blast resistance marker (P_{EF1a}-NLS-dCas9 (N863)-VP64-2A-Blast-WPRE)	Addgene (#61425)
pMF111	Tetracycline-responsive SEAP expression vector ($P_{hCMV*-1}$-SEAP-pA).	Fussenegger, M. et al[1]
pLX78	Lentiviral vector encoding $P_{hCMV*-1}$-driven Gluc expression (LTR-$P_{hCMV*-1}$-Gluc-WPRE-LTR). $P_{hCMV*-1}$-Gluc was PCR-amplified from pLX41 using oligonucleotides OXS205-1 (5`-AGATCCAGTTTGGTTAATTAGCTAGC-3`) and OXS205-2(5`-CGATAAGCTTGATATCTCTAGATTAGTCACCACCGGCCCCCTTGATC-3`) and cloned into the corresponding sequences of pXS205 using the GeneArt Seamless Cloning and Assembly Kit	This work
pXS170	Lentiviral vector encoding $P_{hCMV*-1}$-driven EGFP expression (LTR-$P_{hCMV*-1}$-EGFP-WPRE-LTR). O_{tetO7} was PCR-amplified from pMF111 using oligonucleotides OXS317 (5'-CCCAAGCTTGCCACCATGACGCCCAACAGCACTGGCGAG -3', *Not*I underlined) and OXS316 (5'-TCGAAGCGGCCGGCCTTAGTTCAAGTCCAGGTCGA-CACTGC -3', *Eco*RI underlined), restricted with *Not*I /*Eco*RI and cloned into the corresponding sites (*Not*I /*Eco*RI) of pLL3.7	This work
pXS172	Constitutive P_{hCMV}-driven mammalian expression vector for (P_{hCMV}-2xNLS-CRY2PHR-NLS-VP64). 2xNLS-CRY2PHR-NLS-VP64 was PCR-amplified from pLSC26 using oligonucleotides OXS335 (5'-CTAGTCTAGATAAGCAGAGCTCTCTGGCGCCACCATG -3', *Xba*I underlined) and OXS336 (5'-CCGGAATTCCACCACACTGGACTAGTGGATCCGAGCT -3',	This work

	*Eco*RI underlined), restricted with *Xba*I /*Eco*RI and cloned into the corresponding sites (*Nhe*I /*Eco*RI) of pLL3.7	
pXS173	Constitutive P_{hCMV}-driven mammalian expression vector for TetR-wNES-CIBN (P_{hCMV}-TetR-wNES-CIBN). TetR-wNES-CIBN was excised from pLSC48 with *Nhe*I /*Eco*RI and cloned into the corresponding sites (*Nhe*I /*Eco*RI) of pLL3.7	This work
pXS204	Lentiviral vector encoding constitutive P_{EF1a}-driven mammalian expression units for dCas9 (LTR-$P_{EF1\alpha}$-dCas9-T2A-Blasticidin-LTR). dCas9 was PCR-amplified from was PCR-amplified from Lenti #61425 (Addgene) using oligonucleotides OXS409 (5'-CGGAATTCGTACGGCCACCATGAAAAGGCCGGCGGCCACG -3') and OXS410 (5'-CGGGATCCAGCGGCCGCCACCTTCCTCTTTTTCTTAGGTCCG -3'), restricted with *Eco*RI/*Bam*HI and cloned into the corresponding sites (*Eco*RI/*Bam*HI) of pXS200	This work
pXS205	Lentiviral vector encoding $P_{hCMV*-1}$-driven mammalian expression units for MCP-VPR (LTR-$P_{hCMV*-1}$-MCP-VPR-T2A-Hygro-LTR). MCP-VPR was PCR-amplified from pXS192 using oligonucleotides OXS411 (5'-TGGTTAATTAGCTAGCTCGATACGCGTGCGGCGATGTTTCG -3') and OXS412 (5'-CAGACTTCCTCTGCCCTCGGATCCAAACAGAGATGTGTCGAAGATGGACAG -3'), restricted with *Nhe*I/*Bam*HI and cloned into the corresponding sites (*Nhe*I/*Bam*HI) of pXS201	This work
pXS206	Lentiviral vector encoding constitutive P_{hEF1a}-driven mammalian expression units for CRY2PHR-VP64 (LTR-$P_{EF1\alpha}$-CRY2PHR-VP64-T2A-Puromycin-LTR). CRY2PHR<br>-VP64 was PCR-amplified from pXS188 using oligonucleotides OXS413 (5'-CGGAATTCAGGTCTATATAAGCAGAGCTCTCTGGCGCCACCATGG -3') and OXS414 (5'-CGCGGATCCATCGATATATAACATATCGAGATCG -3'), restricted with *Eco*RI/*Bam*HI, and cloned into the corresponding sites (EcoRI/BamHI) of pXS202	This work
pXS207	Lentiviral vector encoding constitutive P_{hEF1a}-driven mammalian expression units for TetR-CIBN (LTR-$P_{EF1\alpha}$-TetR-CIBN-T2A-Zeocin-LTR). TetR-CIBN was PCR-amplified from pXS183 using oligonucleotides OXS415 (5'-CGGAATTCGCCACCATGTCCAGACTGGACAAGAGCAAAGTCATAAAC -3') and OXS416 (5'-CGGGATCCGATGTAGTCGGTCTTCTCGAGCTCCTTGG -3'), restricted with *Eco*RI/*Bam*HI and cloned into the corresponding sites (*Eco*RI/*Bam*HI) of pXS203	This work

pS3	Lentiviral vector encoding constitutive expression units for sgRNA (*Sox2*) (LTR-P_{U6}-S84-P_{U6}-S136-P_{U6}-S148-LTR). Seed sequence of sgRNA (*Sox2*-84) was PCR-amplified from pSox2-84 using oligonucleotides OLX457 (5`-CGACGCGTCGGAGGGCCTATTTCCCATGATTCC-3`, *Mlu*I underlined) and OLX458 (5`-CTAGCTAGCTAGCAAAAAAAGCACCGACTCGGTGC-3`, *Mlu*I underlined), sgRNA (*Sox2*-136) was PCR-amplified from p Sox2-136 using oligonucleotides OLX455 (5`-CTAGCTAGCTAGGAGGGCCTATTTCCCATGATTCC-3`, *Mlu*I underlined) and OLX459 (5`-CCCAAGCTTGGGCAAAAAAAGCACCGACTCGGTGC-3`, *Hin*dIII underlined), sgRNA (*Sox2*-148) was PCR-amplified from pSox2-148 using oligonucleotides OLX456 (5`-CCCAAGCTTGGGGAGGGCCTATTTCCCATGATTCC-3`, *Hin*dIII underlined) and OLX460 (5`-GGGTCTAGACAAAAAAAGCACCGACTCGGTGC-3`, *Xba*I underlined). All three seed sequences were restricted with *Mlu*I/*Nhe*I, *Nhe*I/ *Hin*dIII, *Hin*dIII/*Xba*I, respectively, and cloned into corresponding sites (*Mlu*I/ *Xba*I) of pXS193	This work
pO2	Lentiviral vector encoding constitutive expression units for sgRNA (*Oct4*) (LTR-P_{U6}-O71-P_{U6}-O127-LTR). Seed sequence of sgRNA (*Oct4*-71) was PCR-amplified from pOct4-71 using oligonucleotides OLX457 (5`-CGACGCGTCGGAGGGCCTATTTCCCATGATTCC-3`, *Mlu*I underlined) and OLX458 (5`-CTAGCTAGCTAGCAAAAAAAGCACCGACTCGGTGC-3`, *Nhe*I underlined), sgRNA (*Oct4*-127) was PCR-amplified from pOct4-127 using oligonucleotides OLX455 (5`-CTAGCTAGCTAGGAGGGCCTATTTCCCATGATTCC-3`, *Nhe*I underlined) and OLX459 (5`-CCCAAGCTTGGGCAAAAAAAGCACCGACTCGGTGC-3`, *Hind*III underlined). Two seed sequences were restricted with *Mlu*I/*Nhe*I, *Nhe*I/ *Hin*dIII, respectively, and cloned into corresponding sites (*MluI/Hin*dIII) of pXS193	This work
pS3-O2	Lentiviral vector encoding constitutive expression units for the concatenated sgRNA (LTR-P_{U6}-S84-P_{U6}-S136-P_{U6}-S148-P_{U6}-O71-P_{U6}-O127-LTR). Seed sequence of sgRNA (*Sox2*) was PCR-amplified from pS3 using oligonucleotides pLX491 (5`-CCGCTCGAGCAAGGCAAGGCTTGACCGACAATTG -3`, XhoI underlined) and PLX493(5`-CTAGCTAGCCTGGCAACTAGAAGGCACAGTCGAG -3`, *Nhe*I underlined), sgRNA (*Oct4*) was PCR-amplified from pO2 using oligonucleotides pLX492 (5`-	This work

	CTAGCTAGCcaaggcaaggcttgaccgacaattg -3`, *Nhe*I underlined) and pLX494 (5`- CGGAATTCCTGGCAACTAGAAGGCACAGTCGAG -3`, *Eco*RI underlined). Two seed sequences were restricted with *Xho*I/ *Nhe*I, *Nhe*I/ *Eco*RI, respectively, and cloned in corresponding sites *Xho*I/ *Eco*RI of pXS193	
pWS74	Lentiviral vector encoding constitutive expression units for sgRNA1 (*NEUROG2*) (LTR-P_{U6}-sgRNA1 (*NEUROG2*)-LTR). Seed sequence of sgRNA1 (*NEUROG2*) was PCR-amplified from pJY251 using oligonucleotides OWS405-1(5'-ctagctagcCGAGGGCCTATTTCCCATGA-3') and OWS405-2 (5'-ccggaattcATACCGCACAGATGCGTAA-3'), restricted with *Nhe*I/*Eco*RI and cloned into the corresponding sites (*Nhe*I/*Eco*RI) of pLL3.7	Shao, et al[2]
pWS76	Lentiviral vector encoding constitutive expression units for sgRNA2 (*NEUROG2*) (LTR-P_{U6}-sgRNA2 (*NEUROG2*) -LTR). Seed sequence of sgRNA2 (*NEUROG2*) was PCR-amplified from pJY254 using oligonucleotides OWS405-1(5'-ctagctagcCGAGGGCCTATTTCCCATGA-3') and OWS405-2 (5'-ccggaattcATACCGCACAGATGCGTAA-3'), restricted with *Nhe*I/*Eco*RI and cloned into the corresponding sites (*Nhe*I/*Eco*RI) of pLL3.7	Shao, et al[2]
pGY174	Constitutive mammalian stable expression vector for sgRNA1 (*Lama1*), sgRNA2 (*Lama1*) and dCas9 (P_{U6}-sgRNA1 (*Lama1*)-pA-P_{U6}-sgRNA2 (*Lama1*)-pA-P_{hCMV}- dCas9-pA). P_{U6}-sgRNA2 (*Lama1*) was PCR-amplified from pGY172 using oligonucleotides OGY168-1 (5'ccggaattcGAGGGCCTATTTCCCATGATTCC-3') and OGY168-2 (5'- cgcggatccATTCAAAAAAGCACCGACTCG-3'), restricted with *Eco*RI/ *Bam*HI and cloned into the corresponding sites (*Eco*RI/ *Bam*HI) of pGY173	Shao, et al[2]
pGY177	Constitutive mammalian stable expression vector for sgRNA1 (*Fst*), sgRNA2 (*Fst*) and dCas9 (P_{U6}-sgRNA1 (*Fst*)-pA-P_{U6}-sgRNA2 (*Fst*)-pA-P_{hCMV}- dCas9-pA). P_{U6}-sgRNA2 (*Fst*) was PCR-amplified from pGY176 using oligonucleotides OGY169-1 (5'-cgggatccCTTTTGCTGGCCTTTTGCTCA-3') and OGY169-2 (5'-cgacgcgtGCGGGTGTCGGGGCTGGCTTAAC-3'), restricted with *Bam*HI/ *Mlu*I and cloned into the corresponding sites (*Bam*HI/ *Mlu*I) of pGY176	Shao, et al[2]
pJY251	Constitutive sgRNA1 (*NEUROG2*) expression vector (PU6-sgRNA1 (NEUROG2)-pA). Seed sequence of sgRNA1 (*NEUROG2*) was generated by annealing oligonucleotides OJY251-1 (5'-caccGTGGATGGCCAGGCCAGGGGA-3') and OJY251-2 (5'-aaacTCCCCTGGCCTGGCCATCCAC-3'), restricted with *Bbs*I and cloned into the corresponding sites (*Bbs*I) of sgRNA (MS2) cloning backbone.	Shao, et al[2]

pJY254	Constitutive sgRNA2 (*NEUROG2*) expression vector (P_{U6}-sgRNA2 (*NEUROG2*)-pA). Seed sequence of sgRNA2 (*NEUROG2*) was generated by annealing oligonucleotides OJY254-1 (5'-caccGCTGCAGCCTGGAGCGCCAAC-3') and OJY254-2 (5'-aaacGTTGGCGCTCCAGGCTGCAGC-3'), restricted with *Bbs*I and cloned into the corresponding sites (*Bbs*I) of sgRNA (MS2) cloning backbone.	Shao, et al[2]
pGY172	Constitutive sgRNA1$_{Lama1}$ and dCas9 expression vector (P_{U6}-sgRNA1 $_{Lama1}$-pA::P_{hCMV}-dCas9-pA).	Shao, et al[2]
pGY173	Constitutive sgRNA1$_{Lama1}$, sgRNA2$_{Lama1}$ and dCas9 expression vector (P_{U6}-sgRNA1$_{Lama1}$-pA::P_{U6}-sgRNA2 $_{Lama1}$-pA::P_{hCMV}-dCas9-pA).	Shao, et al[2]
pGY176	Far-red light induced dCas9(N)-Coh2 fusion protein expression vector expression vector [P_{FRL}-dCas9-Coh2-pA; $P_{FRL,}$ pA-(whiG)$_3$-$P_{hCMVmin}$].	Shao, et al[2]
pLSC26	Constitutive 2×NLS-CRY2PHR- 1×NLS-VP64 expression vector (P_{hCMV}-2×NLS-CRY2PHR-1×NLS-VP64-pA).	This work
pLSC48	Constitutive TetR-CIBN expression vector (P_{hCMV}-TetR-CIBN-pA).	This work
pLX41	Lentiviral vector encoding $_{TetO7\text{-}hCMVmin}$ ($P_{hCMV*\text{-}1}$) driven mammalian expression unit for Gluciferase (Gluc)-insulin [LTR-$P_{TetO7\text{-}hCMV\text{-}min}$ ($P_{hCMV*\text{-}1}$)-Gluc-insulin-LTR].	This work
pOct4-71	Lentiviral vector encoding P_{U6} driven for *Oct4*-71 (O71) (LTR-P_{U6}-O71-LTR).	This work
pOct4-127	Lentiviral vector encoding P_{U6} driven for *Oct4*-127 (O127) (LTR-P_{U6}-O127-LTR).	This work
pSox2-84	Lentiviral vector encoding P_{U6} driven for *Sox2*-84 (S84) (LTR-P_{U6}-S84 -LTR).	This work
pSox2-136	Lentiviral vector encoding P_{U6} driven for *Sox2*-136 (S136) (LTR-P_{U6}-S136 -LTR).	This work
pSox2-148	Lentiviral vector encoding P_{U6} driven for *Sox2*-148 (S148) (LTR-P_{U6}-S148 -LTR).	This work
pXS183	Lentiviral vector encoding constitutive P_{hEF1a}-driven mammalian expression unit for TetR-wNES-CIBN and P_{mPGK}-driven mammalian expression unit for PuroR (LTR - P_{hEF1a}-TetR-wNES-CIBN::mPGK-PuroR-LTR).	This work
pXS188	Lentiviral vector encoding constitutive P_{hEF1a}-driven mammalian expression unit for 2xNLS-CRY2PHR-NLS-VP64 and P_{mPGK}-driven mammalian expression unit for PuroR (LTR - P_{hEF1a} - 2xNLS-CRY2PHR-NLS-VP64::mPGK-PuroR-LTR).	This work
pXS192	Lentiviral vector encoding $_{tetO3g}$ -driven mammalian expression unit for MS2-VPR and P_{mPGK}-driven mammalian expression unit for ZeoR (LTR-P_{tetO3g}-MS2-VPR::mPGK-ZeoR-LTR).	This work

pXS193	Lentiviral vector encoding constitutive P_{U6} -driven mammalian expression unit for sgRNA$_{MS2}$ and constitutive P_{hCMV}-driven mammalian expression unit for EGFP (LTR-P_{U6}-sgRNA$_{MS2}$ ::P_{CMV}-EGFP-LTR).	This work
pXS200	Lentiviral vector encoding constitutive P_{hEF1a}-driven mammalian expression unit for T2A-BlastR (LTR-P_{hEF1a}-T2A-BlastR-LTR).	This work
pXS201	Lentiviral vector encoding constitutive P_{hEF1a}-driven mammalian expression unit for T2A-BlastR (LTR-P_{hEF1a}-T2A- HygroR-LTR).	This work
pXS202	Lentiviral vector encoding constitutive P_{hEF1a}-driven mammalian expression unit for T2A-BlastR (LTR-P_{hEF1a}-T2A-PuroR-LTR).	This work
pXS203	Lentiviral vector encoding constitutive P_{hEF1a}-driven mammalian expression unit for T2A-BlastR (LTR-P_{hEF1a}-T2A-ZeoR-LTR).	This work

Oligonucleotides: Restriction endonuclease-specific sites are underlined in lowercase letters , annealing base pairs are indicated in capital letters, the homologous recombination sequences are underlined in capital letters.

Abbreviations: BL, blue light; **CIBN**, N-terminal fragment of cryptochrome-interacting basic-helix-loop-helix 1; **CRY2**, Cryptochrome 2 in Arabidopsis thaliana; **CRY2PHR,** the photolyase homology region of CRY2PHR; **TetR,** Tet repressor; **dCas9,** nuclease deactivated Cas9; **EGFP**, enhanced green fluorescent protein; **Gluc,** Gaussia Luciferase; ***Fst,*** follistatin gene; **ITR,** inverted terminal repeat; **WPRE:** Woodchuck hepatitis virus posttranscriptional regulatory element; ***Lama1,*** laminin subunit alpha 1 gene; **MCS,** multiple cloning site; **MCP,** MS2 coat protein; **NES**, nuclear export signal; **NLS,** nuclear localization signal; **PCR**, polymerase chain reaction; **P2A**, picornavirus-derived self-cleaving peptide engineered for bicistronic gene expression in mammalian cells; **T2A**, a self-cleaving 2A peptide derived from a virus of the insect thosea asigna; **pA**, polyadenylation signal; **P_{hCMV}**, human cytomegalovirus immediate early promoter; **$P_{hCMV*-1}$**, tetracycline-responsive promoter (tetO_7-$P_{hCMVmin}$); **$P_{hCMVmin}$**, minimal version of P_{hCMV}; **$P_{hEF1\alpha}$**, human extension factor α gene promoter; **P_{U6},** U6 RNA polymerase III promoter; **P_{mPGK}**: Mouse phosphoglycerate kinase 1 gene promoter; **PuroR,** gene product that confers puromycin resistance to mammalian cells; **RNA**, ribonucleic acid; **sgRNA**, single guide RNA; **VP64**, tetrameric core of *Herpes simplex* virus-derived transactivation domain; **VPR,** a tripartite activator VP64-p65-Rta fusion protein; **ZeoR**, gene product that confers zeocin resistance to mammalian cells; **BlastR**, gene product that confers blasticidin resistance to mammalian cells; **HygroR**, gene product that confers hygromycin resistance to mammalian cells.

Table S3. Primers used for qPCR analysis

Gene name	Primer Sequence
Sox2	F: AACGGCAGCTACAGCATGATGC<br>R: CGAGCTGGTCATGGAGTTGTAC
Oct4	F: CAGCAGATCACTCACATCGCCA<br>R: GCCTCATACTCTTCTCGTTGGG
Nanog	F: CCTCCAGCAGATGCAAGAACTC<br>R: CTTCAACCACTGGTTTTTCTGCC
Esrrb	F: CTCGCCAACTCAGATTCGAT<br>R: AGAAGTGTTGCACGGCTTTG
Fgf4	F: CGTGGTGAGCATCTTCGGAGTGG<br>R: CCTTCTTGGTCCGCCCGTTCTTA
Nr5a2	F: ATGGGAAGGAAGGGACAATC<br>R: ATACAAACTCCCGCTGATCG
Lama1	F: TTCCCAGAGGTCTCCATCAATAAC<br>R: GCGCTTGCTTCCTTTACACTCAG
Fst	F: AGGATGTGAACGACAATACTCTC<br>R: ACATTCGTTGCGGTAGGTTTTCC
Pax6	F: TCTTTGCTTGGGAAATCCG<br>R: CTGCCCGTTCAACATCCTTAG
N-cadherin	F: TCCTGATATATGCCCAAGACAA<br>R: TGACCCAGTCTCTCTTCTGC
Tuj1	F: TAGACCCCAGCGGCAACTAT<br>R: GTTCCAGGTTCCAAGTCCACC
Nestin	F: GAATGTAGAGGCAGAGAAAACT<br>R: TCTTCAAATCTTAGTGGCTCC
GAPDH	F: ATGACATCAAGAAGGTGGTG<br>R: CATACCAGGAAATGAGCTTG

Table S4. Target sequences of sgRNAs

**Name**	**Guide Sequence of sgRNA**
***Sox2* locus**	
sgRNA1 (*Sox2*)	GCGAGGCTGGGCTCGGGCGC
sgRNA2 (*Sox2*)	GCTCGGCTCGGCGGCGCGGC
sgRNA3 (*Sox2*)	GCGCTCTGCTGGGCTCGGCT
***Oct4* locus**	
sgRNA1 (*Oct4*)	GGGTGGAGGAGCAGAGCTGT
sgRNA2 (*Oct4*)	AACCTCCGTCTGGAAGACAC
***NEUROG2* locus**	
sgRNA1 (*NEUROG2*)	GTGGATGGCCAGGCCAGGGGA
sgRNA2 (*NEUROG2*)	GCTGCAGCCTGGAGCGCCAAC
***Fst* locus**	
sgRNA1 (*Fst*)	CGCAAATAAGAACT
sgRNA1 (*Fst*)	CAAAGCGGCAGGAG
***Lama1* locus**	
sgRNA1 (*Lama1*)	ACGGCGGGGCTCCCCCTGG
sgRNA2 (*Lama1*)	ACAGACCTGGGCGCAGC

Table S5. Chimera mice generated from $iPSC_{LIRE}$ cells

Mice ID	Generations	Sex	Chimera Mouse
**1**	**F0**	♀	**Yes**
2	F0	♀	No
3	F0	♀	No
4	F0	♀	No
5	F0	♀	No
6	F0	♀	No
7	F0	♀	No
**8**	**F0**	♀	**Yes**
**9**	**F0**	♀	**Yes**
10	F0	♀	No
11	F0	♀	No
**13**	**F0**	♂	**Yes**

Amino acids or DNA sequence information

1. dCas9

MYPYDVPDYASPKKKRKVEASDKKYSIGLAIGTNSVGWAVITDEYKVPSKKFKVLGNTDRHS
IKKNLIGALLFDSGETAEATRLKRTARRRYTRRKNRICYLQEIFSNEMAKVDDSFFHRLEESFL
VEEDKKHERHPIFGNIVDEVAYHEKYPTIYHLRKKLVDSTDKADLRLIYLALAHMIKFRGHFLI
EGDLNPDNSDVDKLFIQLVQTYNQLFEENPINASGVDAKAILSARLSKSRRLENLIAQLPGEK
KNGLFGNLIALSLGLTPNFKSNFDLAEDAKLQLSKDTYDDDLDNLLAQIGDQYADLFLAAKN
LSDAILLSDILRVNTEITKAPLSASMIKRYDEHHQDLTLLKALVRQQLPEKYKEIFFDQSKNGY
AGYIDGGASQEEFYKFIKPILEKMDGTEELLVKLNREDLLRKQRTFDNGSIPHQIHLGELHAIL
RRQEDFYPFLKDNREKIEKILTFRIPYYVGPLARGNSRFAWMTRKSEETITPWNFEEVVDKGA
SAQSFIERMTNFDKNLPNEKVLPKHSLLYEYFTVYNELTKVKYVTEGMRKPAFLSGEQKKAI
VDLLFKTNRKVTVKQLKEDYFKKIECFDSVEISGVEDRFNASLGTYHDLLKIIKDKDFLDNEE
NEDILEDIVLTLTLFEDREMIEERLKTYAHLFDDKVMKQLKRRRYTGWGRLSRKLINGIRDKQ
SGKTILDFLKSDGFANRNFMQLIHDDSLTFKEDIQKAQVSGQGDSLHEHIANLAGSPAIKKGIL
QTVKVVDELVKVMGRHKPENIVIEMARENQTTQKGQKNSRERMKRIEEGIKELGSQILKEHP
VENTQLQNEKLYLYYLQNGRDMYVDQELDINRLSDYDVDAIVPQSFLKDDSIDNKVLTRSDK
NRGKSDNVPSEEVVKKMKNYWRQLLNAKLITQRKFDNLTKAERGGLSELDKAGFIKRQLVE
TRQITKHVAQILDSRMNTKYDENDKLIREVKVITLKSKLVSDFRKDFQFYKVREINNYHHAH
DAYLNAVVGTALIKKYPKLESEFVYGDYKVYDVRKMIAKSEQEIGKATAKYFFYSNIMNFFK
TEITLANGEIRKRPLIETNGETGEIVWDKGRDFATVRKVLSMPQVNIVKKTEVQTGGFSKESIL
PKRNSDKLIARKKDWDPKKYGGFDSPTVAYSVLVVAKVEKGKSKKLKSVKELLGITIMERSS
FEKNPIDFLEAKGYKEVKKDLIIKLPKYSLFELENGRKRMLASAGELQKGNELALPSKYVNFL
YLASHYEKLKGSPEDNEQKQLFVEQHKHYLDEIIEQISEFSKRVILADANLDKVLSAYNKHRD
KPIREQAENIIHLFTLTNLGAPAAFKYFDTTIDRKRYTSTKEVLDATLIHQSITGLYETRIDLSQL
GGDSPKKKRKVEAS

2. MCP

MASNFTQFVLVDNGGTGDVTVAPSNFANGVAEWISSNSRSQAYKVTCSVRQSSAQKRKYTIK
VEVPKVATQTVGGVELPVAAWRSYLNMELTIPIFATNSDCELIVKAMQGLLKDGNPIP

SAIAANSGIY

3. O$_{TetO7}$-P$_{hCMVmin}$

CTCGAGTTTACCACTCCCTATCAGTGATAGAGAAAAGTGAAAGTCGAGTTTACCACTCCCT
ATCAGTGATAGAGAAAAGTGAAAGTCGAGTTTACCACTCCCTATCAGTGATAGAGAAAAG
TGAAAGTCGAGTTTACCACTCCCTATCAGTGATAGAGAAAAGTGAAAGTCGAGTTTACCA
CTCCCTATCAGTGATAGAGAAAAGTGAAAGTCGAGTTTACCACTCCCTATCAGTGATAGAG
AAAAGTGAAAGTCGAGTTTACCACTCCCTATCAGTGATAGAGAAAAGTGAAAGCCTGCAG
GTCGAGCTCGGTACCCGGGTCGAGTAGGCGTGTACGGTGGGAGGCCTATATAAGCAGAGC
TCGTTTAGTGAACCGTCAGATCGCCTGGAGACGCCATCCACGCTGTTTTGACCTCCATAGA
AGACACCGGGACCGATCCAGCCTCCGCG

4. 2xNLS-CRY2PHR-NLS-VP64

PKKKRKVEASASPKKKRKVEASKMDKKTIVWFRRDLRIEDNPALAAAAHEGSVFPVFIWCP
EEEGQFYPGRASRWWMKQSLAHLSQSLKALGSDLTLIKTHNTISAILDCIRVTGATKVVFNH
LYDPVSLVRDHTVKEKLVERGISVQSYNGDLLYEPWEIYCEKGKPFTSFNSYWKKCLDMSIE
SVMLPPPWRLMPITAAAEAIWACSIEELGLENEAEKPSNALLTRAWSPGWSNADKLLNEFIE
KQLIDYAKNSKKVVGNSTSLLSPYLHFGEISVRHVFQCARMKQIIWARDKNSEGEESADLFL
RGIGLREYSRYICFNFPFTHEQSLLSHLRFFPWDADVDKFKAWRQGRTGYPLVDAGMRELW
ATGWMHNRIRVIVSSFAVKFLLLPWKWGMKYFWDTLLDADLECDILGWQYISGSIPDGHEL
DRLDNPALQGAKYDPEGEYIRQWLPELARLPTEWIHHPWDAPLTVLKASGVELGTNYAKPI
VDIDTARELLAKAISRTREAQIMIGAAPASPKKKRKVEASGRAMGSGRADALDDFDLDMLGS
DALDDFDLDMLGSDALDDFDLDMLGSDALDDFDLDMLIN

5. TetR-wNES-CIBN

HHVQTGQEQSHKLCSGITQ*SRYRRPDDKETRSKAGS*AAYPVLAREEQAGPARCPGNRDAG
QASYPLLPPGRRVMARLSAEQRQVIPLCSPLTSRRG*SASRHPPNRETVRNPGKSARVPVSAR
LLPGERTVRSVRRGPLYTGLRIGGSGASSSKRGKRDTYHRFYAPTSETSN*AVRPSGSRTCLPF
RPGTNHMWPGETAKVRKRxLYPERLRRILTNGAIGGDLLLNFPDMSVLERQRAHLKYLNPTF

DSPLAGFFADSSMITGGEMDSYLSTAGLNLPMMYGETTVEGDSRLSISPETTLGTGNFKKRKF
DTETKDCNEKKKKMTMNRDDLVEEGEEEKSKITEQNNGSTKSIKKMKHKAKKEENNFSNDS
SKVTKELEKTDYIGS

6. scFv-GCN4-NLS-P65-HSF1

MGPDIVMTQSPSSLSASVGDRVTITCRSSTGAVTTSNYASWVQEKPGKLFKGLIGGTNNRAPG
VPSRFSGSLIGDKATLTISSLQPEDFATYFCALWYSNHWVFGQGTKVELKRGGGGSGGGGSGG
GGSSGGGSEVKLLESGGGLVQPGGSLKLSCAVSGFSLTDYGVNWVRQAPGRGLEWIGVIWG
DGITDYNSALKDRFIISKDNGKNTVYLQMSKVRSDDTALYYCVTGLFDYWGQGTLVTVSSYP
YDVPDYAGGGGGSGGGGSGGGGSGGGGSPKKKRKVAAAGSPSGQISNQALALAPSSAPVLA
QTMVPSSAMVPLAQPPAPAPVLTPGPPQSLSAPVPKSTQAGEGTLSEALLHLQFDADEDLGAL
LGNSTDPGVFTDLASVDNSEFQQLLNQGVSMSHSTAEPMLMEYPEAITRLVTGSQRPPDPAPT
PLGTSGLPNGLSGDEDFSSIADMDFSALLSQISSSGQGGGGSGFSVDTSALLDLFSPSVTVPDM
SLPDLDSSLASIQELLSPQEPPRPPEAENSSPDSGKQLVHYTAQPLFLLDPGSVDTGSNDLPVLF
ELGEGSYFSEGDGFAEDPTISLLTGSEPPKAKDPTVS

7. MCP-VPR

MASNFTQFVLVDNGGTGDVTVAPSNFANGVAEWISSNSRSQAYKVTCSVRQSSAQKRKYTIK
VEVPKVATQTVGGVELPVAAWRSYLNMELTIPIFATNSDCELIVKAMQGLLKDGNPIPSAIAAN
SGIYSAGGGGSGGGGSGGGGSGDALDDFDLDMLGSDALDDFDLDMLGSDALDDFDLDMLG
SDALDDFDLDMLYIDSSGSPKKKRKVGPSGQISNQALALAPSSAPVLAQTMVPSSAMVPLAQ
PPAPAPVLTPGPPQSLGSGSGSRDSREGMFLPKPEAGSAISDVFEGREVCQPKRIRPFHPPGSPW
ANRPLPASLAPTPTGPVHEPVGSLTPAPVPQPLDPAPAVTPEASHLLEDPDEETSQAVKALREM
ADTVIPQKEEAAICGQMDLSHPPPRGHLDELTTTLESMTEDLNLDSPLTPELNEILDTFLNDEC
LLHAMHISTGLSIFDTSLF

8. **sgRNA backbone:** P_{U6}-Bbs1-Bbs1-gRNA scaffold-U6-terminator

GAGGGCCTATTTCCCATGATTCCTTCATATTTGCATATACGATACAAGGCTGTTAGAGAGAT
AATTGGAATTAATTTGACTGTAAACACAAAGATATTAGTACAAAATACGTGACGTAGAAAG

TAATAATTTCTTGGGTAGTTTGCAGTTTTAAAATTATGTTTTAAAATGGACTATCATATGCTT
ACCGTAACTTGAAAGTATTTCGATTTCTTGGCTTTATATATCTTGTGGAAAGGACGAAACAC
CGGGTCTTCGAGAAGACCTGTTTTAGAGCTAGAAATAGCAAGTTAAAATAAGGCTAGTCC
GTTATCAACTTGAAAAAGTGGCACCGAGTCGGTGCTTTTTTGTTTTAGAGCTAGAAATAGC
AAGTTAAAATAAGGCTAGTCCGTTTTT

Supplementary References

1. M. Fussenegger, R. P. Morris, C. Fux, M. Rimann, B. von Stockar, C. J. Thompson, J. E. Bailey, *Nature biotechnology* **2000**, *18*, 1203-1208.
2. J. Shao, M. Wang, G. Yu, S. Zhu, Y. Yu, B. C. Heng, J. Wu, H. Ye, *Proceedings of the National Academy of Sciences - PNAS* **2018**, *115*, E6722-E6730.
